# Development of Au_*x*_Cu_*y*_Pd_*z*_ Nanocomposites as Therapeutic Agents: Enhancing Cancer Treatment through Autophagy Modulation and Immune-Associated Effects

**DOI:** 10.1021/acsami.5c20536

**Published:** 2026-03-04

**Authors:** Li-Xing Yang, Yi-Chun Chiu, Yi-Lun Chen, Ting-Ying Chen, Yi-Tseng Tsai, Yu-Cheng Chin, Ya-Ling Yeh, Ying-Jan Wang, Chih-Chia Huang, Rong-Jane Chen, Mei-Yi Liao

**Affiliations:** † Department of Photonics, 34912 National Cheng Kung University, Tainan 701, Taiwan; ‡ Center of Applied Nanomedicine and Core Facility Center, National Cheng Kung University, Tainan 701, Taiwan; § Division of Urology, Department of Surgery, Yangming Branch, Taipei City Hospital, Taipei 111, Taiwan; ∥ Department of Urology, College of Medicine and Shu-Tien Urological Research Center, National Yang Ming Chiao Tung University, Taipei 112, Taiwan; ⊥ Department of Health and Welfare, University of Taipei, Taipei 111, Taiwan; # Department of Social and Public Affairs, University of Taipei, Taipei 111, Taiwan; ¶ Department of Applied Chemistry, 63378 National Pingtung University, Pingtung 900, Taiwan; ∇ Department of Environmental and Occupational Health, College of Medicine, National Cheng Kung University, Tainan 704, Taiwan; ○ Department of Medicinal and Applied Chemistry, Kaohsiung Medical University, Kaohsiung 807, Taiwan; ⧫ Department of Food Safety/Hygiene and Risk Management, College of Medicine, National Cheng Kung University, Tainan 704, Taiwan

**Keywords:** alloy nanoparticles, multimetallic nanocomposites, autophagy modulation, ferroptosis, cancer-selective therapy, immunoregulation, metal-based nanomedicine

## Abstract

The development of multimetallic nanoparticles for cancer treatment represents a significant advancement in the field of nanomedicine. We introduce a Cu-templated synthesis method to create Au_*x*_Cu_*y*_Pd_*z*_ hollow nanomicrostructures, wherein gold atoms stabilize copper (Cu) and facilitate the incorporation of palladium (Pd) through oxidation and coreduction processes. These ternary nanocomposites demonstrate enhanced cellular uptake via the copper transporter CTR1/2-mediated pathway and exhibit superior catalytic activity for the reaction of hydrogen peroxide to generate hydroxyl radicals. The presence of both Cu and Pd triggers significant autophagic responses, increases lipid peroxidation, and disturbs copper metabolism, as indicated by the increased expression of autophagy-related proteins and mitochondrial reactive oxygen species, ultimately leading to selective cancer cell death. The synergistic effects of these three metals not only increase autophagy but also promote the degradation of immune escapable proteins, including IDO1, PD-L1, and CD47. Based on the Cu/Pd element-induced biochemical stimulation, we conducted a proof-of-concept in vivo validation using a murine orthotopic bladder tumor model to demonstrate that Au–Cu–Pd ternary nanoparticles enhance autophagy and ferroptosis, thereby reversing the immunosuppressive tumor microenvironment by reducing immune escape proteins. These effects increased the infiltration of antitumor immune cells, with further enhancement from photothermal therapy at a low laser power density and sample dose. Our findings offer valuable insights into designing multimetallic nanoparticles through element chemistry for cancer therapy, highlighting their potential as effective modulators of autophagy.

## Graphical abstract

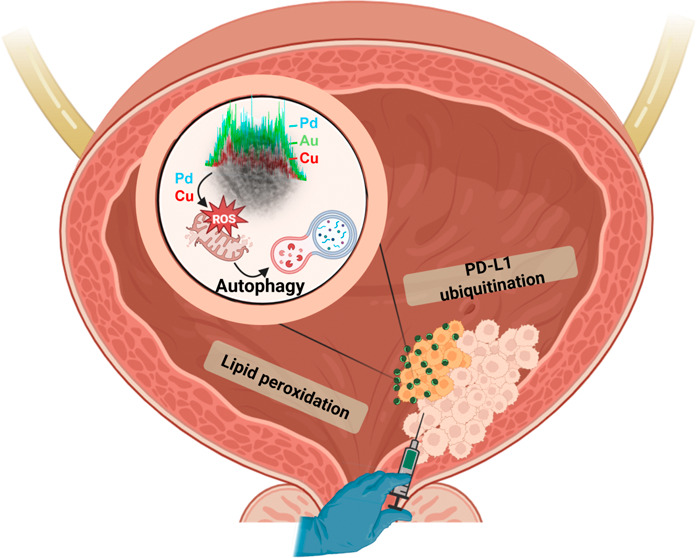

## Introduction

Traditional chemotherapeutic agents often have severe side effects,^1,2^ driving scientists to seek safer and more effective anticancer strategies. The unique mechanisms by which transition metals such as nanozymes^3,4^ and metal-ion-related cell death^5,6^ elicit cascades in tumor disruption by altering the redox balance or metabolism offer promising directions for the development of next-generation anticancer therapeutics.^4^ The multivalent nature of metal species is important in the development of many human diseases, including antioxidant, aging, and neurodegenerative disorders.^7,8^ Transition metal ion regulation is linked to endocytosis, autophagy, and exocytosis involving lysosomes. For nanometal applications in biomedicine,^9,10^ researchers must investigate both the toxic effects of metal nanoparticles on living organisms and how these metals influence biological activities and defense mechanisms.^11,12^ Various nanomaterials, such as silica, silver, copper, palladium (Pd), tellurium, iron, and their oxide nanoparticles, as well as quantum dots and polymeric nanoparticles, can trigger this process.^13–16^ Although autophagy aids in cellular degradation^17^ and energy production, its inhibition can lead to the accumulation of harmful substances, potentially causing apoptosis. Conversely, excessive autophagy activation by nanoparticles may result in autophagic cell death.^18,19^

Investigating the interactions between nanoparticles and cells enhances our understanding of nanotoxicology, which is crucial for developing safer, more effective, and precise nanomedicine treatments. Recent research has focused on how individual metallic elements, such as copper (Cu), stimulate cellular responses.^20,21^ Copper is an essential trace metal that supports various enzymes and is known to induce autophagy through copper oxide nanoparticles (CuO NPs).^22^ Studies by Heonyong Park et al.^23^ have indicated that Cu_2_O particles are more effective than CuO at triggering autophagy, potentially because of the generation of reactive oxygen species (ROS). Furthermore, Cu-dependent AuCu nanoparticles can promote exocytosis in bladder cancer cells, thereby reducing oxidative stress.^24^ Wen et al. reported that nano-CuPd induced autophagy in HeLa cells, which was related to mitochondrial damage by CuPd and the generation of ROS.^25^ However, the fundamental mechanism remains unclear. Palladium (Pd(0)), recognized for its biocompatibility, exhibits promising peroxidase-like catalytic properties^26,27^ in pharmaceuticals and could be a safer alternative to chemical drugs. Research indicates that Pd­(II) complexes have significantly lower cytotoxicity than cisplatin,^28^ suggesting that they are potential new anticancer agents. Additionally, Pd nanoparticles disrupt lysosomal function, impeding autophagic flux and resulting in cell death.^29^ Moreover, tetrapod Pd nanoenzymes can induce autophagy in macrophages to degrade oxidized low-density lipoprotein while enhancing immunotherapy by eliminating immune evasion proteins, such as PD-L1, from cancer cells.^30,31^

Despite advancements in research focused on single metal elements in nanoparticles,^20–23^ a significant gap remains in our understanding of the anticancer effects of multimetallic nanoparticle on cells. Although the combination of multiple elements presents challenges, incorporating target metals into inert crystal structures is promising. Nevertheless, much remains unknown about their catalytic mechanisms within biological systems. In this study, a Cu templating synthesis was developed to fabricate an assembly of Au_*x*_Cu_*y*_Pd_*z*_ nanoparticles, which formed hollow nanomicrostructures. The Au atoms in the ternary nanoalloy-stabilized Cu allow Pd atoms to be incorporated more easily through oxidation and coreduction with Cu and Au. In addition, the specialized transport system associated with Cu^21,32^ and the cellular engulfment properties of Cu-based nanoparticles enable the efficient delivery of therapeutic substances into cells, effectively triggering subsequent biological responses. The gradual release of metals from nanoalloys helps reduce the risk of acute death in normal cells, providing a safer alternative to traditional chemotherapies, such as doxorubicin (DOX) and cisplatin. The incorporation of Cu/Pd into the ternary nanocomposites increased intracellular catalytic activity to produce ROS-mediated autophagic responses and upregulated the expression of Cu metabolism-related genes (Scheme 1). The Au_*x*_Cu_*y*_Pd_*z*_ nanocomposite induced autophagy over time, enhancing the degradation of immune escape proteins such as IDO1, PD-L1, and CD47.^33^ In vivo data support its ability to convert a cold tumor into a hot one and boost the therapeutic efficacy against bladder tumors when combined with photothermal treatment (Scheme 1). These results suggest that the Au–Cu–Pd nanocomposite is a promising immunomodulatory agent and a safer alternative to conventional anticancer drugs, making it a strong candidate for the development of new nanomedicine.

**Scheme 1:**
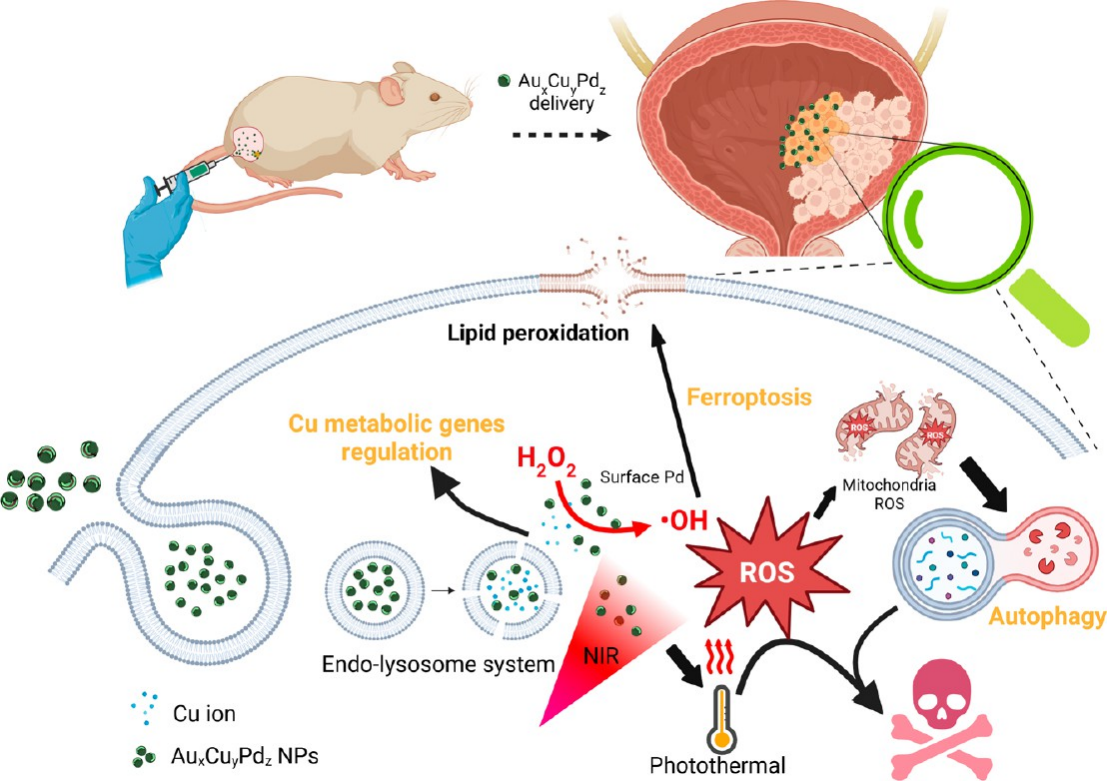
Schematic Illustration Showing that Pd-Doped AuCu NPs Enhance Cell Uptake and Induce ROS-Mediated Mitochondrial Damage, Resulting in Autophagy and Ultimately Leading to Cell Death^a^

a An additional photothermal effect, applied by NIR irradiation, can further enhance tumor growth inhibition in the murine orthotopic bladder cancer model.

## Materials and Methods

### Materials

Gold­(III) chloride trihydrate (HAuCl_4_, Sigma-Aldrich), copper­(II) chloride dihydrate (CuCl_2_·2H_2_O, J.T. Baker), palladium chloride (PdCl_2_, Sigma-Aldrich), hydrogen peroxide (Sigma-Aldrich), poly­(styrene-*alt*-maleic acid) sodium salt solution (PSMA, Sigma-Aldrich), hydrazine hydrate (N_2_H_4_·H_2_O, Alfa Aesar), hexadecyl trimethylammonium bromide (CTAB, Sigma-Aldrich), l-ascorbic acid (AA, J.T. Baker), 3,3′,5,5′-tetramethylbenzidine (TMB, Sigma-Aldrich), 4-nonylphenol (4-NP, Acros Organics), 4-aminophenol (4-AP, Alfa Aesar), 3-(4,5-dimethylthiazol-2-yl)-2,5-diphenyltetrazolium bromide (MTT, GoldBio), dimethyl sulfoxide (DMSO, Fisher Scientific), Chloroquine (CQ, Sigma-Aldrich), Zinc chloride (ZnCl_2_, Sigma-Aldrich), tetrathiomolybdate (TM, Sigma-Aldrich), rapamycin (Merck), Ferrostatin-1 (Merck), MG-132 (MedChemExpress), and cell medium (RPMI-1640, McCoy’s 5A, F-12K, purchased from Corning) were used.

### Characterization

Transmission electron microscopy (TEM) (Hitachi 7500 and JEM-2100F) was used to determine the structures of the nanomaterials. The Au_*x*_Cu_*y*_Pd_*z*_ NP solution on a grid coated with a hole-containing carbon support film was blotted dry with filter paper to leave a thin film of a particle suspension in the wells. The grid was immersed in liquid ethane, cooled by liquid N_2_. Fluorescence spectroscopy (Biotek Synergy H1) and a UV–visible spectrophotometer (JASCO V-730, Japan) were used to measure the fluorescence and absorption of the Au_*x*_Cu_*y*_Pd_*z*_-related samples. Atomic absorption spectroscopy (AAS, SensAA GBC, Australia) was used to measure the metal composition of the Au_*x*_Cu_*y*_Pd_*z*_ NPs. X-ray photoelectron spectroscopy (XPS; PHI 5000 VersaProbe, Japan) was used to measure Au_*x*_Cu_*y*_Pd_*z*_-based NPs with a Mg Kα source (12 kV and 10 mA). The binding energy scale was calibrated to the central C 1s peak at 284.5 eV. The particle sizes and zeta potentials of Au_*x*_Cu_*y*_Pd_*z*_ dispersed in aqueous solution were measured by a dynamic light scattering (DLS) analyzer (Horiba SZ-100, Japan).

### Synthesis of Cu@PSMA NPs

Cu@PSMA was synthesized by mixing 2.5 mL of deionized water, 4 mL of CuCl_2_ solution (5 mM), and 2 mL of PSMA (8.73 mM) in a Florence flask and heating with magnetic stirring for 10 min in an oil bath at 80 °C. A total of 137.5 μL of N_2_H_4_ (20 mM) was then added to the flask, and the mixture was heated with stirring for 2 h. The product was then collected by centrifugation and purified 2 times with deionized water. Finally, the Cu NPs were dispersed in deionized water.

### Syntheses of Au_*x*_Cu_*y*_Pd_*z*_ Nanocomposites

For the synthesis of the Au_*x*_Cu_*y*_Pd_*z*_ nanostructure, we mixed 875 μL of CTAB (0.1426 M), 125 μL of HAuCl_4_ solution (5 mM), 250 μL of AA (0.1 M), and 50 μL of PdCl_2_ (0 5, 5, 10, and 20 mM) with 1000 μL of Cu NP solution (0.86 mM) by stirring at room temperature. After 30 min of reaction, Au_*x*_Cu_*y*_Pd_*z*_ was centrifuged at 4500 rpm for 10 min to separate Au_*x*_Cu_*y*_Pd_*z*_ from the supernatant. All centrifugations were performed more than 3 times, and the samples were washed with D.I. water. In the Pd-free synthesis, Au_72_Cu_28_ nanoshells were fabricated.

### Syntheses of Au_86_Cu_14_ Nanoshells

For the synthesis of the Au_86_Cu_14_ nanoshells, we mixed 3500 μL of CTAB (0.1426 M), 500 μL of HAuCl_4_ solution (5 mM), and 1000 μL of AA (0.1 M) with 500 μL of a Cu NP solution (0.86 mM) by stirring at room temperature. After 30 min of reaction, the Au_86_Cu_14_ nanoshells were centrifuged at 4500 rpm for 10 min to separate the product from the supernatant. All centrifugations were performed more than 3 times, and the samples were washed with D.I. water.

### Catalysis of 4-NP with Au_*x*_Cu_*y*_Pd_*z*_ NPs

Sodium borohydride dissociates to form the boron hydride anion (BH_4_^–^), which is a strong reductant that provides hydrogen atoms that reduce the hydroxyl group (−OH) of 4-nonylphenol (4-NP) to form 4-aminophenol (4-AP) with the amino group (−NH_2_). The surface of Au_*x*_Cu_*y*_Pd_*z*_ effectively facilitates the transfer of electrons from the reducing agent to the 4-NP molecule, thus accelerating the reduction reaction. In a 4 mL quartz cell, 2 mL of an aqueous solution containing NaBH_4_ (0.125 mM) and 4-NP (0.35 mM) and 10 μL of Au_*x*_Cu_*y*_Pd_*z*_ (0.01 mM) were added. The peak absorption at 400 nm was measured every 60 s, and the peak absorption over time was recorded.

### TMB Catalysis for the Measurement of Hydroxyl Radical Production

TMB was used as an indicator for kinetic analysis via colorimetry to investigate the catalysis of the Fenton reaction. The peroxidase-like activity was examined in a quartz cell with 2.28 mL of an aqueous solution containing 0.1 mM Au_*x*_Cu_*y*_Pd_*z*_ and TMB (0.277 mM). Then, 120 μL of a 20 mM H_2_O_2_ solution was added to the above mixed solution, resulting in a final concentration of 1 mM. The absorption peak of TMB at 652 nm was measured every 60 s and recorded over time.

### In Vitro Photothermal Performance of Au_*x*_Cu_*y*_Pd_*z*_

A 0.2 mL portion of Au_*x*_Cu_*y*_Pd_*z*_ solution with different concentrations (0.1, 0.3, and 1 mM) was added to a 96-well transparent plate, followed by irradiation with a 808 nm laser light at a power density of 0.5, 0.75, or 1 W/cm^2^ for 10 min. The solution temperature increment was measured using a T-type thermocouple thermometer with a thermocouple wire to determine the NIR response and photothermal conversion.

### In Vitro Cell Viability Measurement

T24 (human bladder cancer cells), MB49 (mouse bladder cancer cells), and SV-HUC1 (human normal bladder cells) cells were cultured in McCoy’s 5A, RPMI, and F-12K media supplemented with 10% fetal bovine serum and 1% penicillin (P/S) in a 5% CO_2_-filled incubator at 37 °C. The cells were seeded in a 96-well cell culture plate (8000 cells/well) overnight; treated with 100 μL of media containing different concentrations of Au_*x*_Cu_*y*_Pd_*z*_ for 4, 8, 12, and 24 h; and maintained in a 37 °C incubator with 5% CO_2_. In the inhibitor experiments, cells were cotreated with Au_*x*_Cu_*y*_Pd_*z*_ and inhibitors (CQ, ZnCl_2_, TM, rapamycin, ferrostatin-1) at the indicated concentration for 24 h. After the media containing the NPs were removed, 100 μL of MTT reagent (1.2 mM) was added to both the control and test groups. The samples were then placed in an incubator for another hour to obtain purple crystals formed by the reaction between the assay and the mitochondria of live cells. The purple crystals were dissolved in DMSO, and the absorbance was measured with a microplate reader (Synergy H1, BioTek) at 565 nm. The viability of the cells was then calculated.

### Cellular Uptake Measurement

T24 cells (2 × 10^5^ cells) were cultured with Au_72_Cu_28_ or Au_36_Cu_5_Pd_59_ NPs (0.025 mM) in a six-well culture plate for 6 h, after which the culture media was replaced with fresh culture media, and incubation was continued for 6 and 24 h. The cells were rinsed with phosphate-buffered saline (PBS) 3 times, harvested with 0.25% trypsin, and centrifuged at 1000 rpm for 10 min to obtain cell pellets. The precipitated cells were dissolved in 4.5 M HCl and then subjected to inductively coupled plasma–mass spectrometry (iCAP 7400 ICP-OES, Thermo) to determine the concentration of Au taken up by the cells.

### In Vitro Release Assay of Au_36_Cu_5_Pd_59_ NPs

Au_36_Cu_5_Pd_59_ nanoparticles (4 mM) were dispersed in deionized water and PBS (pH 4 and pH 7) and incubated for 1 h, 4 h, 24 h, 3 days, and 7 days. At each time point, 1 mL of the supernatant from each condition was collected by centrifugation at 10,000 rpm for 10 min. The concentrations of released Au, Cu, and Pd were then quantified using ICP (ELEMENT XR, Thermo) analysis.

### Autophagy Analysis

Acridine orange (AO) was used to detect autophagy in the cells. The internal AO dye that accumulates in acidic vesicles produces a red fluorescence signal, and an increase in red fluorescence indicates the occurrence of autophagy.^34^ T24 and MB49 cells were treated with Au_72_Cu_28_ or Au_36_Cu_5_Pd_59_ NPs (0.01, 0.025, and 0.05 mM) for 8 and 24 h, respectively. Then, AO (10 μg/mL) in the serum-free medium was added for 20 min of staining. After staining, the cells were washed with PBS and then analyzed by flow cytometry (FACS Calibur, BD Biosciences).

### Confocal Microscopy for Autophagy Imaging

We used antibodies (*anti*-LC3 and anti-Lamp-2) to label the autophagosome marker protein LC3 (green fluorescence) and the lysosome marker protein Lamp-2 (red fluorescence). DAPI staining (5 μg/mL) was used to visualize the cell nuclei (blue fluorescence). Cells treated with different Au_36_Cu_5_Pd_59_ NPs were fixed with 4% paraformaldehyde, incubated with antibodies and DAPI for 60 min, and then imaged with a confocal microscope system (Zeiss LSM780).

### Cell Death Analysis

The percentage of apoptotic cells was measured by Annexin V and propidium iodide (PI) staining (Annexin V-FITC Apoptosis Detection Kit; BD Biosciences). T24 and MB49 cells were cultured in a 6-well plate (2 × 10^5^ cells per well) and harvested at 8 and 24 h after being treated with Au_72_Cu_28_ or Au_36_Cu_5_Pd_59_ NPs (0.01, 0.025, and 0.05 mM). The harvested cells were stained with Annexin V and PI for 30 min and measured via a flow cytometer (FACS Calibur, BD Biosciences). Early apoptotic cells maintain an intact cell membrane, which prevents PI from penetrating and generating a red fluorescence signal. Annexin V, which has a high affinity for phosphatidylserine exposed on the inner membrane of apoptotic cells, selectively stains early apoptotic cells. Late apoptotic cells, characterized by a loss of membrane integrity, are positive for both PI and Annexin V staining.

### Lipid Peroxidation Analysis

BODIPY 581/591 C11 (4,4-difluoro-5-(4-phenyl-1,3-butadienyl)-4-bora-3a,4a-diaza-s-indacene-3-undecanoic acid) was used to detect lipid peroxidation in cells. T24 and MB49 cells were treated with Au_72_Cu_28_ or Au_36_Cu_5_Pd_59_ NPs for 16 and 24 h, respectively. Then, BODIPY 581/591 C11 (10 μM) in serum-free medium was added for 40 min. After staining, the cells were washed with PBS and then analyzed by flow cytometry (FACS Calibur, BD Biosciences).

### Mitochondrial ROS, Intracellular Iron Content, and Mitophagy Assessment

In vitro mitochondrial ROS generation was determined by the mitochondrial superoxide detection probe MitoSox red (catalog no. M36007, Thermo Fisher Scientific, Inc.). T24 and MB49 cells were cultured in 96-well μCLEAR black plates (catalog no. 655090, Greiner Bio-One GmbH) and treated with Au_72_Cu_28_ or Au_36_Cu_5_Pd_59_ NPs for 1, 2, or 4 h. Then, MitoSox red (5 μM), MitoTracker green (0.5 μM, cat no. M7514, Thermo Fisher Scientific, Inc.) and Hoechst 33342 (1 μg/mL, cat no. H3570, Thermo Fisher Scientific, Inc.) were added to the serum-free medium. After 30 min of incubation, the cells were washed with PBS before imaging with an ImageXpress Confocal HT.ai high-content imaging system (Molecular Devices, LLC). For intracellular iron content measurement, the cells were stained with FerroOrange (0.5 μM, cat no. F374, DOJINDO Laboratories) in serum-free medium for 30 min and then imaged with a high-content imaging system. For mitophagy detection, the cells were stained with a Mitophagy Detection Kit (cat no. MD01, DOJINDO Laboratories) according to the manufacturer’s instructions and then subjected to a high-content imaging system (Molecular Devices, LLC).

### Western Blot Assay

T24 and MB49 cells cultured in 6-well dishes (2 × 10^5^ per well) subjected to 0.01, 0.025, and 0.05 mM Au_72_Cu_28_ or Au_36_Cu_5_Pd_59_ NP treatments (w/o CQ and MG-132) for 8 and 16 h were harvested and lysed in lysis buffer (M-PER mammalian protein extraction reagent, Thermo Fisher Scientific, Inc.) for 30 min on ice and then centrifuged at 14,000 rpm for 20 min at 4 °C to remove precipitates. The obtained proteins were adjusted to equal loads (20 μg per well). The proteins were separated via electrophoresis on a 12% sodium dodecyl sulfate–polyacrylamide gel and subsequently transferred to a 0.45 μm polyvinylidene difluoride (PVDF) membrane, which was blocked with 5% skim milk and immunoblotted with NRF2 (cat no. #A0674, ABclonal, Inc.), *anti*-GPX4 (cat no. #ab125066, 1:3000 dilution, Abcam plc.), LC3B (cat no. #3868, 1:3000 dilutions, Cell Signaling Technology, Inc.), *anti*-IDO1 (cat no. #GTX634652, 1:1000 dilutions, GeneTex, Inc.), *anti*-PD-L1 (cat no. #GTX104763, 1:1000 dilutions, GeneTex, Inc.), anti-CD47 (cat no. #GTX132762, 1:2000 dilutions, GeneTex, Inc.), ubiquitin (cat no. #646302, BioLegend, Inc.), and GAPDH (cat no. #2118, 1:5000 dilutions, Cell Signaling Technology, Inc.) monoclonal antibodies. The membranes were then washed with Tris-buffered saline supplemented with 0.1% Tween-20 and incubated again with a horseradish peroxidase (HRP)-conjugated secondary antibody (catalog no. #7074, 1:5000 dilution; Cell Signaling Technology, Inc.). The corresponding bands were detected via an HRP substrate (Merck Millipore) and captured via an imaging system (UVP Bio-Spectrum; Analytik Jena US LLC, Upland, CA). The images were analyzed by ImageJ software (NIH, Bethesda, MD) for protein expression normalization and quantification.

### Immunoprecipitation (IP) Assay

T24 cells were seeded in 10 cm dishes (1 × 10^6^ cells per dish) and treated with 0.05 mM Au_36_Cu_5_Pd_59_ NPs for 16 or 24 h, followed by a harvest and lysis process in lysis buffer on ice for 30 min. Lysates were clarified by centrifugation at 14,000 rpm for 20 min at 4 °C to remove insoluble debris, and the supernatants were collected. The total protein concentration was adjusted, and 200 μg of protein was incubated with 1 μg of *anti*-PD-L1 antibody (cat. no. #66248-1-Ig, Proteintech) for 24 h at 4 °C. Protein A-coated beads (cat. no. #GE17-5280-01, Merck KGaA) were then added to capture the antigen–antibody complexes at 4 °C for 1 h, followed by washing and protein elution according to the manufacturer’s instructions to obtain the immunoprecipitated PD-L1. The immunoprecipitated PD-L1 was subsequently analyzed by Western blot and probed with an antiubiquitin antibody (cat. no. #646302, BioLegend, Inc.) to determine the ubiquitination level.

### RNA Isolation and Real-Time PCR Quantification (qPCR)

Total RNA was extracted via TRIzol Reagent (Invitrogen), and qPCR was conducted on a StepOnePlus real-time PCR instrument (Thermo Fisher Scientific, Inc.) with SYBR Green reagent (KAPA). The expression of the target gene was normalized to the expression of glyceraldehyde-3-phosphate dehydrogenase (GAPDH). The sequences of primers used for human and mouse quantification are listed in Supporting Information Tables S1 and S2, respectively.

### Murine Orthotopic Bladder Cancer Model

The 12 week-old C57BL/6 female mice were purchased from the Laboratory Animal Center, Medical College, National Cheng Kung University (Tainan, Taiwan), and all research protocols of the animal study were approved by the Institutional Animal Care and Use Committee (IACUC) of the Laboratory Animal Center of National Cheng Kung University Medical College (approval no. 111341). An orthotopic bladder-cancer-bearing mouse model was established by in situ injection with MB49 cancer cells (1 × 10^6^ cells suspended in 100 μL of PBS) into the bladder wall. The tumor-bearing mice were randomly divided into 5 groups (*n* = 4/group) and treated with (1) in situ injections of PBS on days 1, 4, 8 and 11; (2) in situ injections of Au_86_Cu_14_ (a Pd-free control with a closer Au/Cu ratio), or (3) Au_36_Cu_5_Pd_59_ NP (1 mM, 100 μL) for 1 h on Days 1, 4, 8, and 11. An additional photothermal therapy (808 nm laser with 750 mW/cm^2^ for 10 min) was performed after (4) Au_86_Cu_14_ or (5) Au_36_Cu_5_Pd_59_ NP (1 mM, 100 μL) for 1 h on days 1, and 8. The tumor growth and body weight of each mouse were recorded every 7 and 2 days, respectively. The tumor area of each mouse was measured by ultrasound (US) and analyzed with ultrasonic software (VisualSonics). On day 21, the mice were sacrificed, and the major organs (heart, liver, spleen, lungs, and kidneys) and tumors were removed and preserved in a 4% paraformaldehyde solution for histological analysis. For the photothermal therapy group, the mice were sacrificed on day 14, and the tumors were isolated for histological analysis.

### Histological Examination and Immunohistochemistry (IHC) Staining

Paraffin-embedded mouse tissue sections (heart, liver, spleen, lungs, kidneys, and tumors) were stained with hematoxylin and eosin. The H&E-stained sections were then visualized by a microscope (at 20× objective). The tumor sections of all treatment groups were subjected to IHC staining (DAB500 Merck Millipore) to determine protein levels in tumor tissues. IHC staining-related primary antibodies included PD-L1 (cat no. #66248-1-Ig, 1:500 dilution, Proteintech), CD47 (cat no. #20305-1-AP, 1:500 dilution, Proteintech), IDO1 (cat no. #13268-1-AP, 1:500 dilution, Proteintech), CD8 (cat no. #29896-1-AP, 1:500 dilution, Proteintech), CD80 (cat no. #PA5-85913, 1:500 dilution, Invitrogen), LC3 (cat no. #PM036, 1:1000 dilution, MBL), GPX4 (cat no. # A25009, 1:1000 dilution, ABclonal), and Ki67 (cat no. #A2094, 1:100 dilution, ABclonal). Images were captured by a microscope at 200× magnification.

### Statistical Analysis

Statistical analysis was performed using GraphPad Prism 7.0 software. Student’s *t*-test was used to compare two groups to assess significant differences (treatment group vs control group). Each experimental group consisted of a minimum of three independent samples to ensure the robustness and reproducibility of the findings. Statistical significance is denoted as follows: *p < 0.05, **p < 0.01, ***p < 0.001, and ****p < 0.0001.

## Results and Discussion

### Synthesis and Characterization of Au_*x*_Cu_*y*_Pd_*z*_ Nanoshells

Figure 1a presents the UV–visible spectra, which reveal that the dominant absorption band at approximately 590 nm from the Au_72_Cu_28_ nanoshells prepared with 125 μL of HAuCl_4_ at a concentration of 5 mM exhibits a decrease in intensity due to the damping effect associated with surface plasmon resonance when subjected to sequential additions of PdCl_2_ solution at concentrations of 5 mM, 10 mM, and 20 mM. Figure 1b–e shows TEM images of the central region of the Au_*x*_Cu_*y*_Pd_*z*_ material, which is characterized by a core-free nanostructure at varying concentrations of PdCl_2_. These findings indicate the formation of multilayered stacking nanoparticles composed of numerous small particles (∼6.1 nm) on the outer shell to form a micronanoshell structure. The development of these multilayers is highly contingent upon increasing concentrations of PdCl_2_, resulting in an increase in overall particle size from 41.7 ± 6.9 nm at 0 mM PdCl_2_ (Figure 1b) to 67.7 ± 7.4 nm at 20 mM PdCl_2_ (Figure 1e), with intermediate sizes of 43.3 ± 8.2 nm at 5 mM (Figure 1c) and 44.8 ± 7.3 nm at 10 mM (Figure 1d). Hydrodynamic diameter analysis showed a slight increase in particle sizes, as indicated by DLS measurements (Table S3). This suggests that the surface coating of the PSMA polymer enhances its ability to attract water molecules, resulting in larger particle sizes in the liquid phase compared to those in their dry form, as observed via TEM. Atomic absorption spectroscopy measurements (Figure 1f) confirmed that the population of Pd within the Au_*x*_Cu_*y*_Pd_*z*_ nanoshells increased following the sequential interaction of the Cu nanotemplate with both Au and Pd ions. The oxidation of Cu atoms in the Au_*x*_Cu_*y*_ nanostructure is proposed to play a pivotal role in enhancing the binding of Pd atoms to the particle surface. This process occurs because of the reduction in the Cu content rather than the substitution of Au, as indicated by the data presented in the Au and Cu plots (Figure S1).

**Figure 1:**
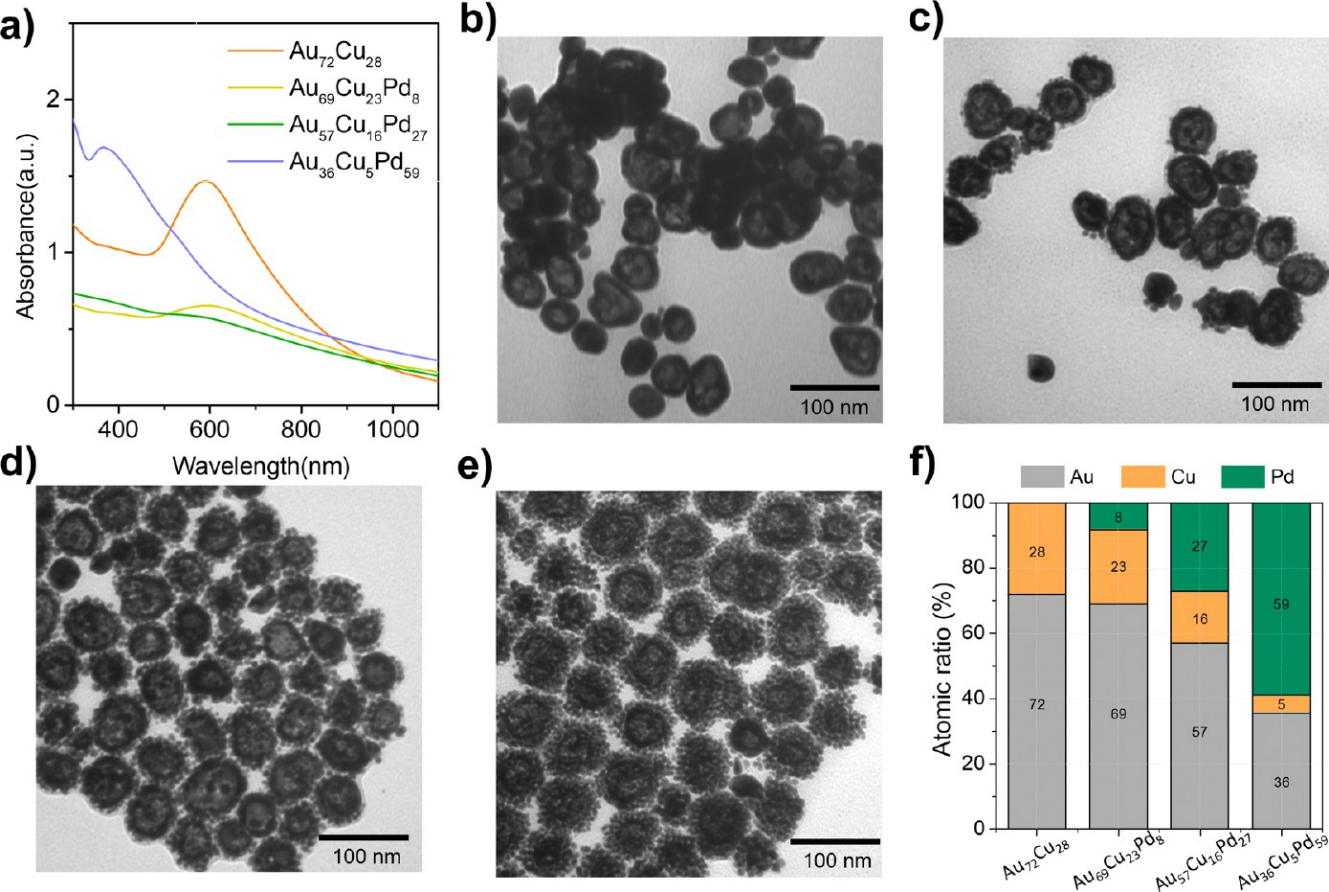
Material characterization of Au_*x*_Cu_*y*_Pd_*z*_@PSMA nanoparticles with different Pd ratios (0–20%): (a) UV–Visible spectra of as-prepared Au_*x*_Cu_*y*_Pd_*z*_ nanoparticles and TEM images of (b) Au_72_Cu_28_, (c) Au_69_Cu_23_Pd_8_, (d) Au_57_Cu_16_Pd_27_, and (e) Au_36_Cu_5_Pd_59_ nanocomposites. (f) AAS elemental analysis of Au_*x*_Cu_*y*_Pd_*z*_ nanoparticles.

The high-resolution TEM (HR-TEM) image accompanied by EDS mapping analysis presented in Figure 2a was employed to investigate the microstructures of the Au_36_Cu_5_Pd_59_. The analysis revealed a homogeneous distribution of Au within the nanoparticles, which was colocalized with Pd and Cu atoms inside the nanoshells. Additionally, the Au/Cu/Pd atoms were observed on the surface of the tiny nanoparticles and at a lower concentration within the interior of a single particle based on the EDS-integrated line scan analysis (Figure 2b). These results suggest that the substitution of the Cu atoms in the AuCu nanoshells by the Au and Pd ions may initiate a coreduction process with the Cu ions, leading to the formation of ternary alloy nanoparticles. The growth of the ternary nanoparticles occurs at a slow rate. A monolayer coating develops within 5 min, followed by multilayer deposition between 10 and 30 min (Figure S2). However, in the absence of HAuCl_4_ ions, the reaction resulted in porous nanoshells (Figure S3a–c). This finding indicates that the integration of Au atoms enhances the structural robustness and integrity of shell-like nanostructures while preventing excessive oxidation of Cu nanoparticles (Figure S3b–d).

**Figure 2:**
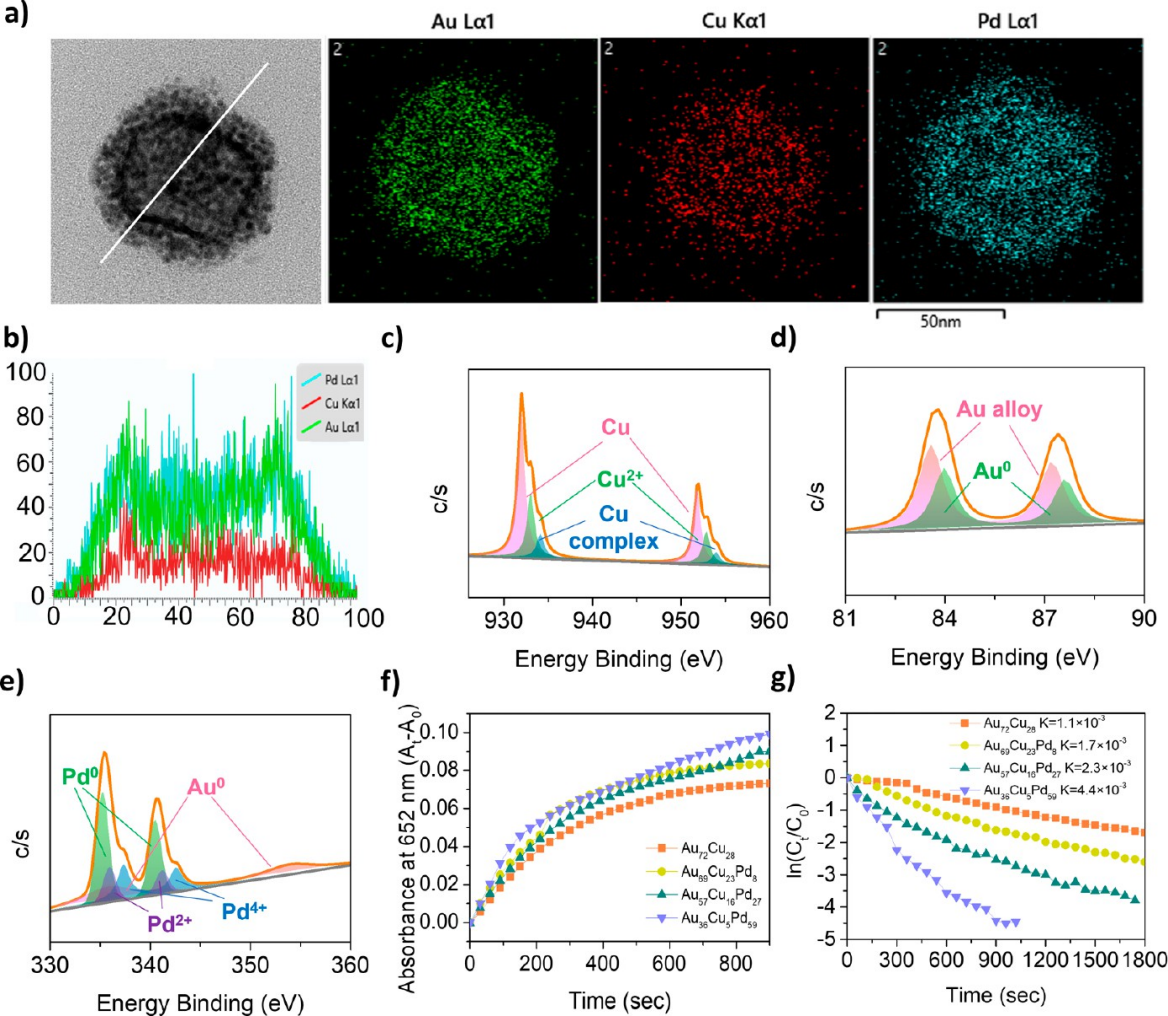
(a) High-magnification TEM image of the Au_36_Cu_5_Pd_59_ nanoparticles and corresponding EDS mapping images and (b) line-scan analysis of Au, Cu, and Pd. XPS spectra for the binding energies of (c) Cu 2p, (d) Au 4f, and (e) Pd 3d + Au 4d by the Au_36_Cu_5_Pd_59_ nanoparticles. UV–visible records for the (f) catalytic reduction of 4-NP and (g) catalytic oxidation of TMB by the Au_72_Cu_28_, Au_69_Cu_23_Pd_8_, Au_57_Cu_16_Pd_27_, and Au_36_Cu_5_Pd_59_ nanoparticles.

In previous investigations,^24^ Au_72_Cu_28_ and Au_86_Cu_14_ nanoshells were synthesized by adjusting HAuCI_4_-based growth solution volumes to react with Cu nanoparticles (approximately 55 nm). The bimetallic Au_72_Cu_28_ nanoshells present a relatively high Cu content within the Au_*x*_Cu_*y*_ nanostructure, structural stability, and favorable dispersion characteristics in aqueous solutions, enhancing their ability to reduce Pd­(II) ions. However, the deposited Pd-related structures displayed heterogeneous growth alongside the Au_*x*_Cu_*y*_ nanostructures rather than causing structural destruction of the particles. Notably, a 7.1% difference in the lattice constants occurs with Cu at 361.5 pm and Pd at 389.1 pm. In comparison, the difference between Au at 407.8 pm and Pd at 389.1 pm is only 4.8%. We propose that the inclusion of Au within the template helps alleviate internal stress in the lattice structure when Pd atoms are deposited by a galvanic replacement reaction^24,35^ into the Au–Cu core and tiny adhered particles from the coreduction with Au and Cu ions form.

In the context of the nanozyme reaction^3,4^ properties of Au_*x*_Cu_*y*_Pd_*z*_ particle assemblages, enhanced interfacial catalytic capability is anticipated due to the increased abundance of Cu and Pd atoms on the surface of Au_*x*_Cu_*y*_Pd_*z*_ structures. To elucidate these properties, we employed XPS to characterize the micronanoshells. Notably, the Au_36_Cu_5_Pd_59_ configuration exhibited a greater ratio of Pd atoms than did the Au_69_Cu_23_Pd_8_ and Au_57_Cu_16_Pd_27_ micronanoshells (Figures S4 and S5). Figures 2c and S5 illustrate the coexistence of Cu(0), CuO, and Cu­(II) complex species at the nanoparticle surfaces of Au_*x*_Cu_*y*_Pd_*z*_. The formation of high oxidation states of Cu in the surface structure is likely attributed to reactions with molecular oxygen during the purification process conducted in an aerobic environment^24,36^ as the coordination of dissolved Cu­(II) ions with the carboxylate groups within the PSMA capping layer occurs.^37,38^ Furthermore, palladium in its zero oxidation state (Pd(0)) was detected across all Au_*x*_Cu_*y*_Pd_*z*_ samples (with *z* >0). The signals observed at elevated binding energy levels for the Pd 3d region increased proportionally with the concentration of Pd atoms in the Au_*x*_Cu_*y*_Pd_*z*_ micronanoshells, indicating the possible formation of Pd­(II) and Pd­(IV) oxides^39^ or complex species with PSMA.^37,38^ The measurement of zeta potential indicated a slight increase in surface charge values, moving from −56.5 mV for Au_72_Cu_28_ to −31.3 mV for Au_69_Cu_23_Pd_8_, and finally to −18.7 and −11.3 mV for Au_57_Cu_16_Pd_27_ and Au_36_Cu_5_Pd_59_, respectively (Table S3). This finding supports the idea that metal ions shield the negative charge of the carboxylate group on the surface of the PSMA polymer coating the particles. With respect to the Au 4f orbitals (Figures 2d and S5), all the analyzed configurations of the Au_*x*_Cu_*y*_Pd_*z*_ micronanoshells exhibited Au(0)-related spectral bands at 84.0 eV (Au 4f_7/2_) and 88.0 eV (Au 4f_5/2_).^40,41^ However, an increased population and a downshift in the binding energy values to 83.8 and 87.4 eV were observed as the number of Pd atoms increased. The binding energies of the Au 4f levels (Figures 2e and S5) demonstrated a downshift correlated with the increased presence of Pd ions. Consequently, in comparison with those of Pd-free AuCu nanoshells, the emergence of Pd 3d signals at high binding energies cannot be overlooked, as they suggest that alloy formation facilitates electron transfer from Pd atoms to Au and Cu at the particle surface, which is attributable to their considerable differences in electronegativity.^37,38,42^ The oxidation states of the different Au, Cu, and Pd atoms in the Au_*x*_Cu_*y*_Pd_*z*_ NPs are summarized in Table S4.

### Enhanced Interfacial Catalytic Activities of Au_*x*_Cu_*y*_Pd_*z*_ Nanoshells

We utilized nitrophenol as a model probe to evaluate the surface reactivity of Au_*x*_Cu_*y*_Pd_*z*_ micronanoshells (see Figures 2f and S6). Our findings indicated that varying the configuration of Pd atoms resulted in the rapid conversion of nitro groups in 4-NP to amino groups in 4-AP within just 15 min. In contrast, micronanoshells with lower Pd contents failed to achieve complete conversion. Upon determining the kinetic constant (*k*), we found that the Au_36_Cu_5_Pd_59_ micronanoshells presented a remarkable rate constant of 4.4 × 10^–3^ s^–1^, significantly surpassing the 1.1 × 10^–3^ s^–1^ rate constant noted for the Au_*x*_Cu_*y*_ nanoalloy. The multilayer assembly of these tiny particles in the Au_36_Cu_5_Pd_59_ micronanoshells not only provided a high surface-to-volume ratio but also introduced numerous reactive sites from the exposed Au and Pd atoms, which served as effective catalytic centers. This arrangement facilitated enhanced charge transfer from the alloy particle interface to the adsorbed molecules during the catalytic reaction.

Furthermore, we conducted the TMB → oxTMB reaction with the addition of H_2_O_2_ (1 mM), a common intracellular biomolecule that helps maintain the oxidation-reduction balance,^43^ in the presence of Au_*x*_Cu_*y*_Pd_*z*_ micronanoshells (Figure 2g). The high concentration of Pd atoms in the Au_*x*_Cu_*y*_Pd_*z*_ micronanoshells resulted in a rapid TMB oxidation conversion rate. Using the Michaelis–Menten equation, we found that the Au_36_Cu_5_Pd_59_ micronanoshells presented the highest *V*_max_ at 0.8992 μM s^–1^ and the lowest *K*_m_ at 2599.407 μM s^–1^ compared with the other Au_*x*_Cu_*y*_Pd_*z*_ micronanoshells. This enhanced performance can be attributed to the efficient electron transfer facilitated by the peroxidase activity associated with the Pd­(II)-^27^ and Pd­(IV)-rich^26^ tiny particles (Figure 2a and Table S4) in the Au_*x*_Cu_*y*_Pd_*z*_ micronanoshells, particularly in relation to the oxidation effects of H_2_O_2_, which generates hydroxyl radicals.

### Au_36_Cu_5_Pd_59_-Induced Cytotoxicity and Autophagy in Bladder Cancer Cells

The ability to modulate ROS levels in cancer cells can potentially prolong cellular damage and further amplify the over activation of autophagy and ferroptosis. This may establish a synergistic strategy for cancer treatment through combination therapy that leverages metal-ion-induced autophagy and chemodynamic therapy (CDT) by inducing redox imbalance. To validate this concept, we conducted toxicity assessments on Au_*x*_Cu_*y*_Pd_*z*_ micronanoshells in bladder cancer cells. Among materials with varying Pd ratios, Au_36_Cu_5_Pd_59_ demonstrated notably stronger cytotoxicity effects (Figure 3a) toward T24 cells, significantly reducing cell viability to 44.5% at a concentration of 0.01 mM. This T24 cytotoxicity effect is Pd concentration-(Figure 3a) and time-dependent (Figure 3b,c). Our results shown in Figure S7 reveal that human normal bladder cells (SV-HUC-1) exhibited higher tolerance to Au_*x*_Cu_*y*_Pd_*z*_ nanoparticles than T24 bladder cancer cells, indicating that these NPs are less harmful to normal bladder cells (Figure 3a). Specifically, SV-HUC-1 cells maintained a 80.3% viability at a concentration of 0.025 mM and retained normal cell morphology. These findings indicate that a higher proportion of Pd enhances the cytotoxic effects on cancer cells while conferring some resistance in normal cells, resulting in superior selectivity over traditional chemotherapy drugs such as cisplatin and doxorubicin^2^ (see Figure S8). As a result, we selected Au_36_Cu_5_Pd_59_ to further explore its anticancer effects on autophagic responses. Owing to the enhanced cytotoxicity by Au_36_Cu_5_Pd_59_ micronanoshells with a high Pd ratio (Figure 3a), we investigated whether this effect was mediated by autophagy leading to cell death, using AO staining to detect acidic autolysosomes that accumulated during the autophagic process. Our results showed that Pd-free AuCu nanoshells did not induce significant autophagy after 8 h (Figure 3d,e). However, the Au_36_Cu_5_Pd_59_ micronanoshells induced autophagy up to 40% in T24 cancer cells after 8 h of treatment, suggesting a potential correlation between the cytotoxicity (Figure 3a,b) of cancer cells and the Pd-mediated autophagic process. In contrast, in normal SV-HUC-1 cells, Au_36_Cu_5_Pd_59_ did not induce a significant induction of autophagy (Figure S9).

**Figure 3:**
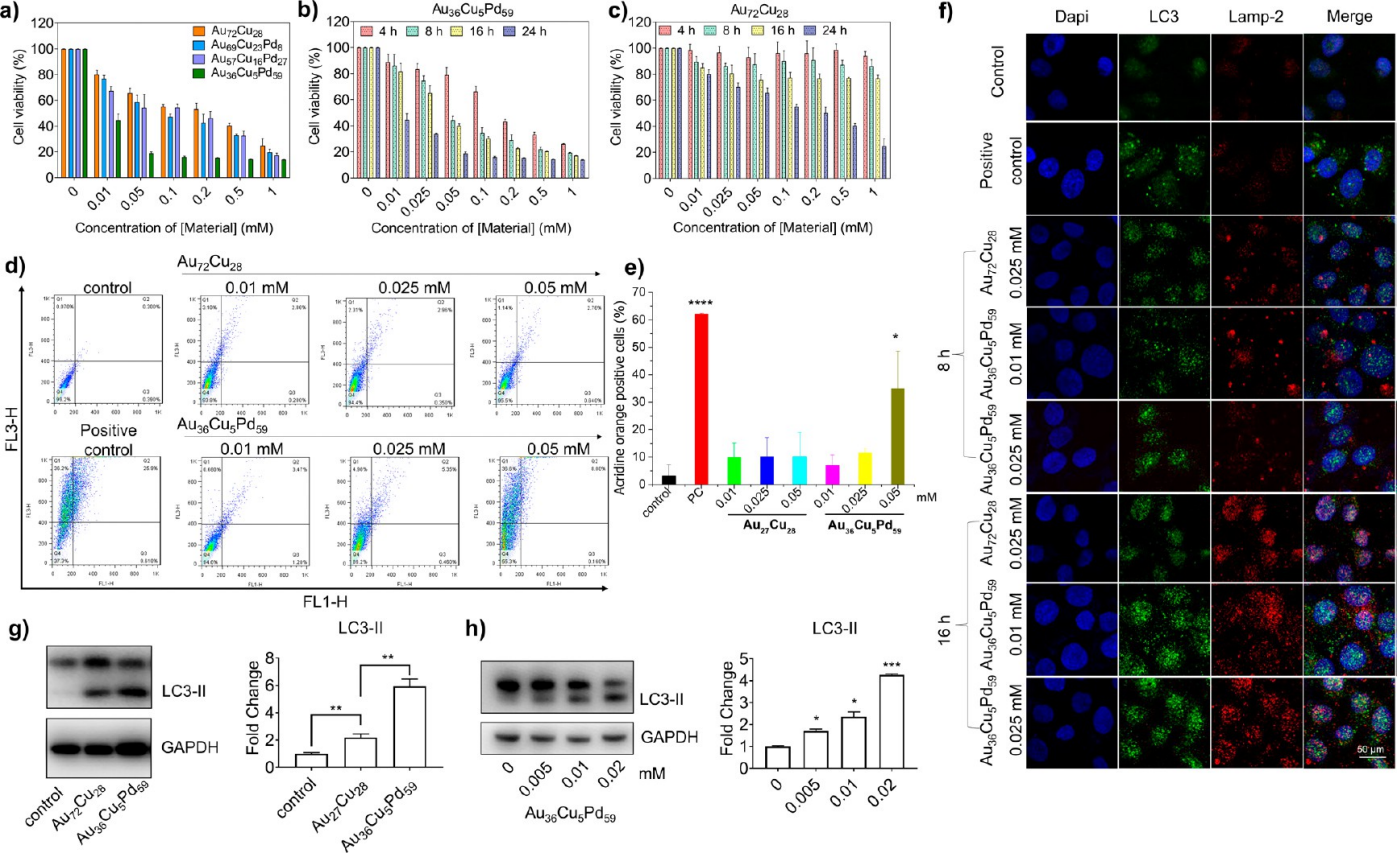
Cytotoxicity and autophagy induction effects of Au_*x*_Cu_*y*_Pd_*z*_ nanoparticles with different Pd ratios on bladder cancer cells. MTT assay with (a) Au_*x*_Cu_*y*_Pd_*z*_ nanoparticles at 24 h and (b,c) different incubation times in T24 human bladder cancer cells. (d) Au_72_Cu_28_ and Au_36_Cu_5_Pd_59_ differentially induced autophagy in T24 human bladder cancer cells at different concentrations at 8 h, as determined via AO staining and flow cytometry analysis. The results were statistically analyzed, with three replicates shown in (e). (f) Confocal microscopy images of T24 cells after treatment with different concentrations of Au_72_Cu_28_ or Au_36_Cu_5_Pd_59_ for 24 h, followed by LC3 and Lamp-2 immunofluorescence staining. Scale bar = 50 μm. (g) Changes in the expression levels of the autophagy protein LC3 in T24 cells after being treated with 0.02 mM Au_72_Cu_28_ or Au_36_Cu_5_Pd_59_ for 24 h, along with the quantification results. (h) Changes in the expression levels of the autophagy protein LC3 in T24 cells after being treated with different concentrations of the Au_36_Cu_5_Pd_59_ micronanoshells for 24 h, along with the quantification results.

Figure 3f shows the results of the immunofluorescence staining, which revealed an increase in both the LC3 and Lamp-2 fluorescence signals, with colocalization evident through yellow signals in the cancer cells, indicating autophagy induction following treatment with Au_36_Cu_5_Pd_59_ micronanoshells. Combined with the flow cytometry analysis presented in Figure S9, this suggests that Au_36_Cu_5_Pd_59_ enhances autophagy in cancer cells while not harming normal cells.

We further conducted Western blot analysis to detect protein expression in T24 cells after treatment for 24 h of treatment. The results showed that the presence of Pd in the Au_36_Cu_5_Pd_59_ micronanoshells induced a higher levels of the autophagy protein LC3-II compared to either the Au_72_Cu_28_ nanocomposite (Figure 3g) or the Au_86_Cu_14_ (Figure S10), which has a similar Au/Cu ratio to Au_36_Cu_5_Pd_59_. This finding suggests that Pd incorporation led to levels of autophagy that were higher than those observed with Au_*x*_Cu_*y*_ NPs. Additionally, Au_36_Cu_5_Pd_59_ micronanoshells induced autophagy in a dose-dependent manner (Figure 3h). Co-treatment with the autophagy flux inhibitor CQ and Au_36_Cu_5_Pd_59_ micronanoshells led to a further increased accumulation of autophagic structures (Figure S11), consistent with concomitant activation of autophagy initiation and blockade of late autophagosome-lysosome fusion and degradation. These results indicate a positive correlation, supporting the role of these micronanoshells in inducing autophagy in T24 cancer cells.

### ROS-Mediated Ferroptosis Triggered by Au_36_Cu_5_Pd_59_ Micronanoshells

Flow cytometric analysis utilizing Annexin V and PI was performed to examine the induction of apoptosis, as illustrated in Figure 4a. The findings revealed that exposure to a low concentration of Au_36_Cu_5_Pd_59_ micronanoshells (0.01 mM) did not significantly increase the percentage of apoptotic cells, which remained below 10%. In contrast, a higher concentration of 0.05 mM led to a more pronounced increase in the percentage of apoptotic cells, reaching approximately 20% (Figure 4b). However, the overall level of apoptosis remained relatively low during the treatment period. Because only a portion of cell death occurs via the apoptotic pathway, we proposed that other mechanisms of cell death require further exploration.

**Figure 4:**
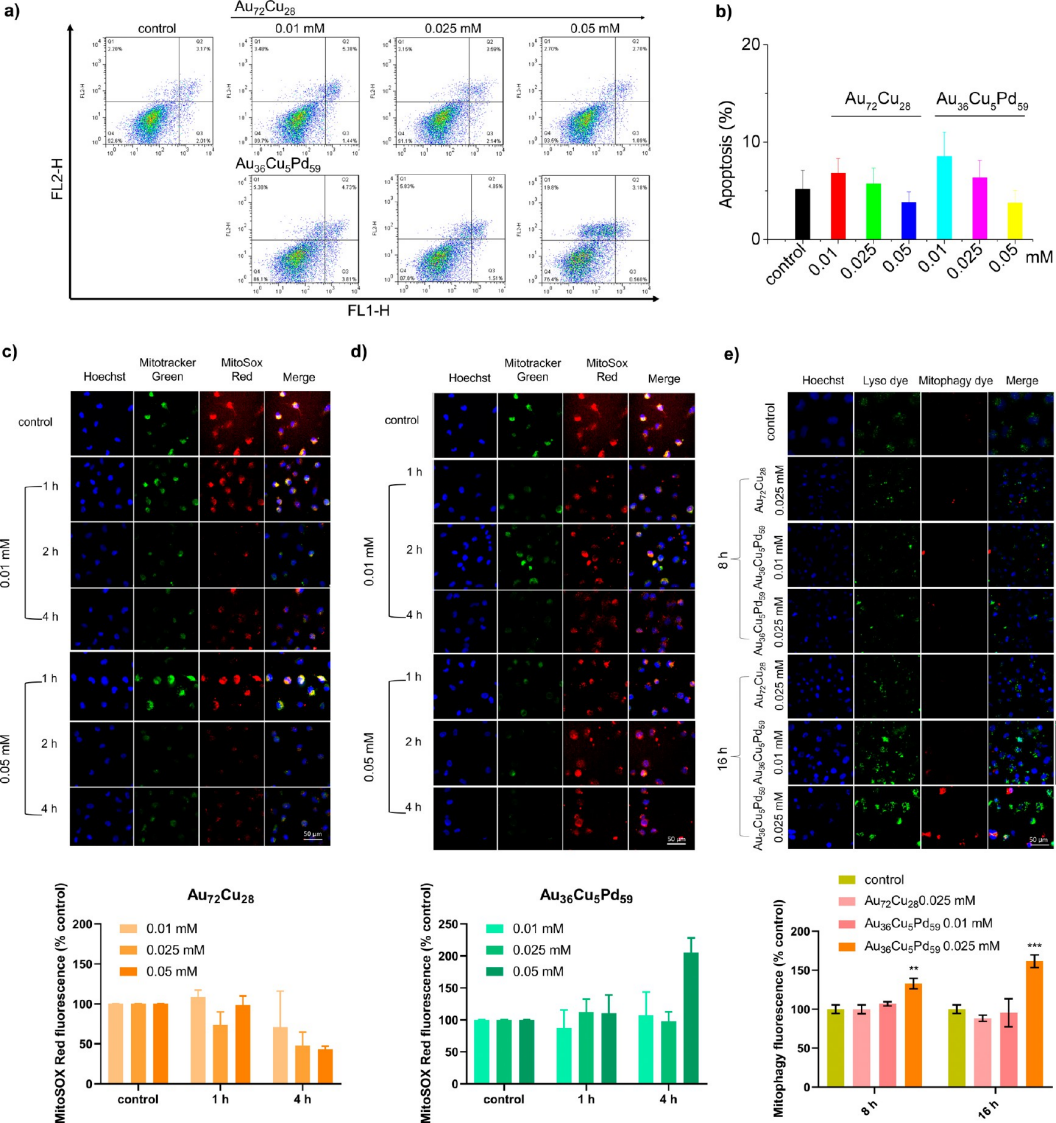
Cell death type analysis. (a) Apoptosis results of cancer cells treated with different concentrations of Au_72_Cu_28_ and Au_36_Cu_5_Pd_59_ NPs via flow cytometric analysis of T24 cells after 4 h of culture. (b) Statistical analysis of apoptosis ratios from triplicate experiments in (a). Fluorescence images of mitochondrial ROS in cancer cells treated with 0.01 and 0.05 mM (c) Au_72_Cu_28_ and (d) Au_36_Cu_5_Pd_59_ NPs in T24 cells after 1, 2, and 4 h of culture with different concentrations of Au_*x*_Cu_*y*_Pd_*z*_ and the related quantitation results (in the below). Scale bar = 50 μm. (e) Mitophagy fluorescence images of T24 cells treated with different concentrations of Au_*x*_Cu_*y*_Pd_*z*_ for 8 and 16 h of culture were obtained with a mitophagy detection kit and the related quantitation results. Scale bar = 50 μm.

Given the catalytic electron transfer properties of AuCu nanocomposites and Pd-based AuCu nanostructures (Figure 2f,g) to small molecules, we assessed the generation of mitochondrial superoxide in Au_*x*_Cu_*y*_Pd_*z*_ nanoparticle-treated cells. Pd-free NPs (Au_72_Cu_28_) induced only slight mitochondrial superoxide generation during the first hour, which quickly diminished in T24 cells (Figure 4c). Pd-containing micronanoshells (Au_36_Cu_5_Pd_59_) induced strong and cumulative mitochondrial superoxide generation at a 0.05 mM concentration within 4 h (Figure 4d), which was consistent with the increased production of ROS resulting from the high reactivity of the Pd-based AuCu nanocomposite with intracellular H_2_O_2_.^44^ We further confirmed that mitophagy was also induced in Au_36_Cu_5_Pd_59_-treated cells at 16 h (Figure 4e). This can be attributed to the progression of mitochondrial damage caused by oxidative stress, triggering subsequent mitophagy.

Furthermore, we investigated the cell death pathway and whether an additional pathway involving ROS-induced lipid peroxidation, possibly through ferroptosis, contributes to cell death. The lipid oxidation staining results indicate that T24 cells exhibit an increase in lipid oxidation levels after prolonged treatment with Au_36_Cu_5_Pd_59_ micronanoshells for 16 h, whereas this phenomenon was not observed in the Au_72_Cu_28_ treatment groups (Figure 5a,b). In addition, the GPX4 protein level also significantly decreased in the Au_36_Cu_5_Pd_59_-treated T24 cells at 16 h (Figure 5c) as well as NRF2 (Figure S12), indicating that the antioxidant activity of T24 cells for removing lipid peroxides was attenuated, which would also lead to lipid peroxidation. These results suggest that peroxidase catalysis by Pd­(II)/Pd­(IV)-rich^26,27^ Au_36_Cu_5_Pd_59_ NPs enhances cellular lipid peroxidation, leading to a similar pathway to iron-induced ferroptotic cell death.^45,46^ The results also explain the relatively low degree of apoptosis observed in Figure 4a. In addition, it has been reported that GPX4 can be degraded via the autophagy process;^47,48^ thus Au_36_Cu_5_Pd_59_ NP-induced autophagy (Figure 3d–f) may also contribute to the downregulation of GPX4 and promote the ferroptotic cell death. Moreover, Figure 5d,e illustrates an increase in iron accumulation in T24 cells treated with the Au_36_Cu_5_Pd_59_ micronanoshells, which was assessed via analysis of intracellular labile iron via a specific ferrous fluorescent probe, FerroOrange. The ferroptosis response induced by Au_36_Cu_5_Pd_59_ micronanoshells is correlated with the intracellular iron concentration, which is likely modulated by the activation of lysosomes. This process ultimately alters the downstream metabolism, leading to elevated levels of intracellular labile iron and increased ferroptosis.

**Figure 5:**
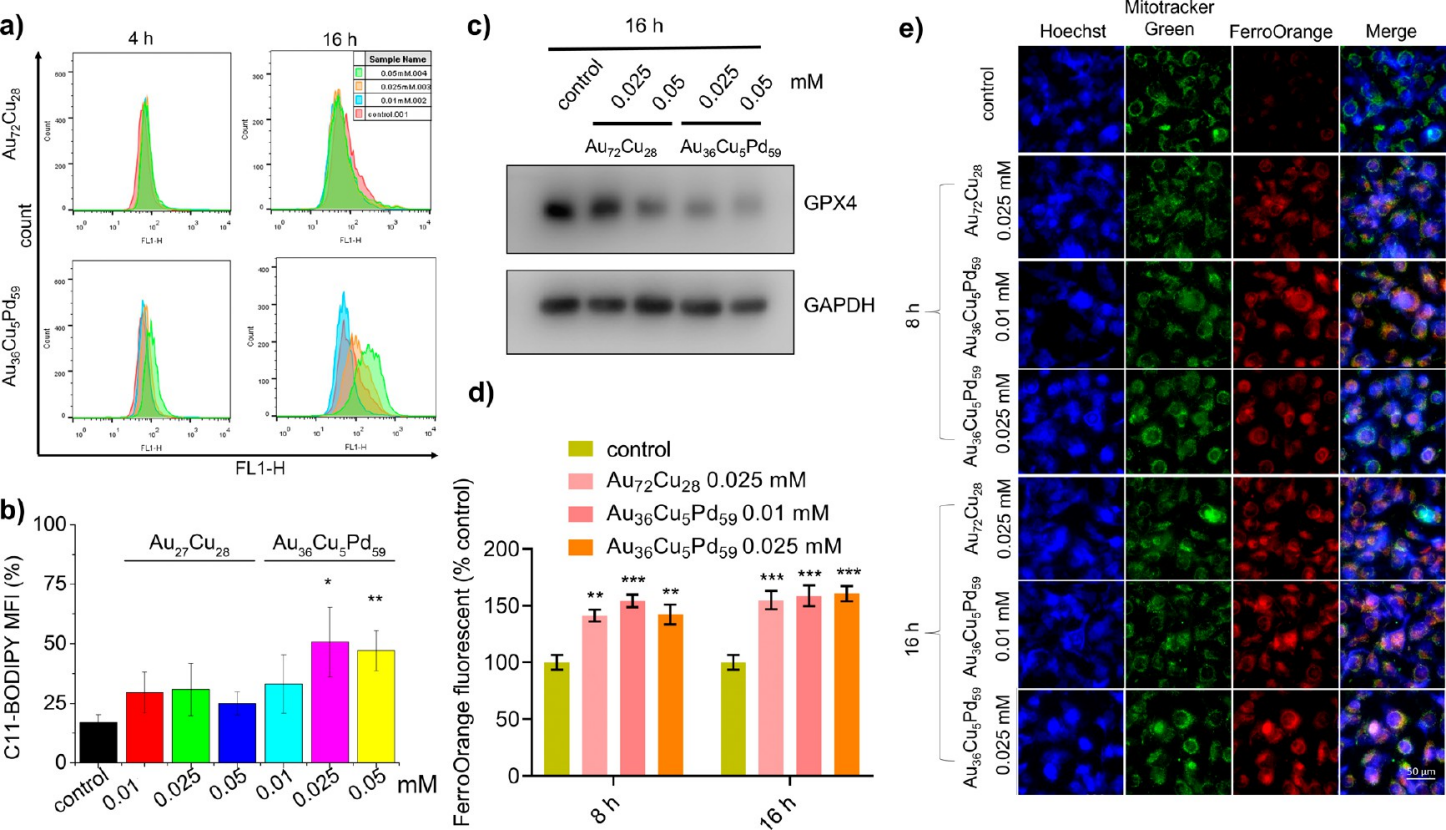
(a) LPO activity in T24 cells treated with different concentrations of Au_72_Cu_28_ or Au_36_Cu_5_Pd_59_ NPs after 4 and 16 h of culture. (b) Statistical analysis of LPO ratios from triplicate experiments in (a). *n* = 3. (c) Changes in the expression levels of GPX4 in T24 cells after coculture with indicated concentrations of Au_72_Cu_28_ or Au_36_Cu_5_Pd_59_ NPs for 16 h, as determined by Western blotting. (d) The quantification results and (e) fluorescent images of intracellular labile iron level analysis in T24 cells treated with Au_*x*_Cu_*y*_Pd_*z*_ NPs for 8 and 16 h by FerroOrange staining, Scale bar = 50 μm.

### Cellular Uptake and Accumulation of Au_*x*_Cu_*y*_Pd_*z*_ in Cancer Cells

We next utilized dark-field microscopy to examine whether Au_*x*_Cu_*y*_Pd_*z*_ NPs are internalized by cells. T24 cancer cells were treated with 0.025 mM Au_72_Cu_28_ or Au_36_Cu_5_Pd_59_ NP at various time points: 2, 6, 8, 16, and 24 h. As shown in Figure 6a, Au_36_Cu_5_Pd_59_ micronanoshells initially adhered to the cell surface, with the majority of light scattering signals observed surrounding the cell periphery at the 2 h mark. As the treatment time increased, the number of punctate signals within the cells also increased (Figure 6b), indicating the continuous uptake of the Au_36_Cu_5_Pd_59_ micronanoshells. In contrast, the uptake of Au_72_Cu_28_ was relatively low and decreased after 16 h. Moreover, a significant increase in uptake in the Au_36_Cu_5_Pd_59_-treated cells compared with those treated with Au_72_Cu_28_ (Figure 6c) was confirmed through Au measurements via atomic absorption spectroscopy during the first 6 h. Notably, following the replacement of the material-containing media with fresh culture media, the Au concentration in the Au_36_Cu_5_Pd_59_-treated cells decreased after 6 to 24 h but remained higher than that in the cells treated with the non-Pd counterpart. These findings suggest that the Au_36_Cu_5_Pd_59_ micronanoshells effectively enter cells and trigger relatively high levels of exocytosis.

**Figure 6:**
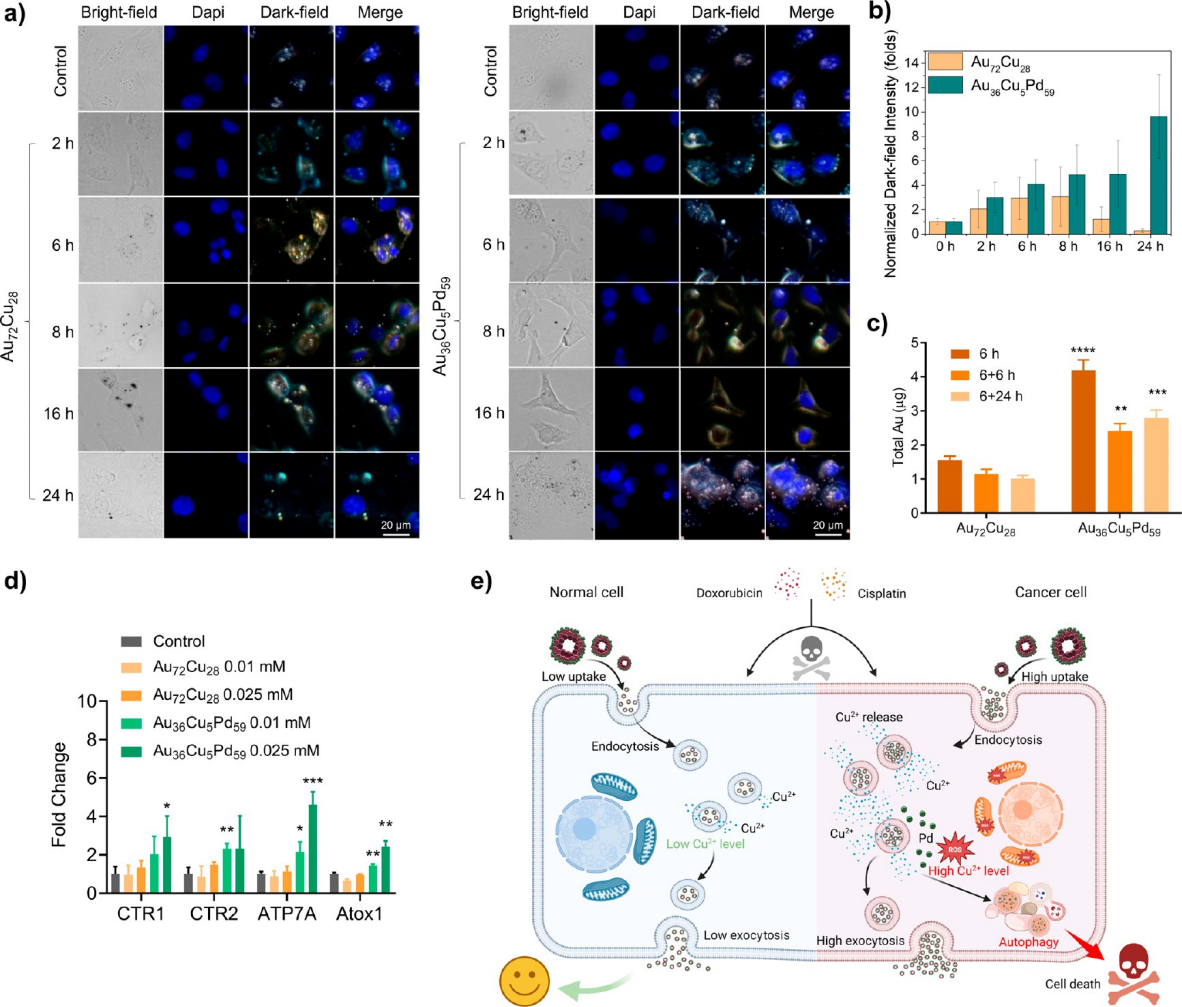
(a) Cell uptake of Au_*x*_Cu_*y*_Pd_*z*_ NPs was measured via dark-field microscopy images of T24 cells treated with 0.025 mM Au_72_Cu_28_ or Au_36_Cu_5_Pd_59_ NPs at different time points (0–24 h). Scale bar = 20 μm. (b) Normalized punctate signals within the cells from (a). (c) Total uptake of Au in T24 cells by atomic absorption spectroscopy during the first 6 h of incubation with Au_*x*_Cu_*y*_Pd_*z*_ micronanoshells, followed by an additional 6 and 24 h after removing the NPs. (d) Cu metabolism-related gene expression analysis of T24 treated with Au_*x*_Cu_*y*_Pd_*z*_ materials for 24 h via qPCR. (e) Schematic illustration showing that Pd-doped AuCu NPs enhanced cell uptake and exocytosis while they still induced ROS and autophagy, leading to cell death. The statistics are compared between Au_72_Cu_28_ and Au_36_Cu_5_Pd_59_ NPs at the same time points.

The regulation of Au_36_Cu_5_Pd_59_ nanoshell uptake and export may be influenced by the Cu metabolism process.^21^ Therefore, we analyzed the expression of genes related to the Cu metabolism in NP-treated cells. In our assessment of Cu metabolism-related gene expression, materials containing Pd (Au_36_Cu_5_Pd_59_) had a more significant effect on the regulation of cellular Cu metabolism than materials devoid of Pd (Au_72_Cu_28_). A substantial increase in the expression of genes responsible for Cu uptake, specifically CTR1/2, was observed in T24 cells treated with Au_36_Cu_5_Pd_59_ micronanoshells (Figure 6d)_._ This finding is consistent with the results of cellular uptake assessed through darkfield imaging (Figure 6a,b) and atomic absorption spectroscopy (Figure 6c). These observations provide evidence that the increased uptake of Au_36_Cu_5_Pd_59_ micronanoshells is mediated by the Cu-related transporter protein CTR1/2. Conversely, the expression of genes associated with Cu transport (Atox1) and Cu efflux (ATP7A/B) also increased in T24 cells treated with Au_36_Cu_5_Pd_59_ micronanoshells (Figure 6d). This finding indicates that after 24 h of incubation with Au_36_Cu_5_Pd_59_ micronanoshells, the sustained accumulation of NPs initiates a cellular defense mechanism that transports and excretes Cu to prevent cell death. However, this mechanism does not efficiently eliminate the NPs, resulting in significant NP accumulation and cell death.

Conversely, normal SV-HUC cells exhibited significant expulsion of Au_*x*_Cu_*y*_Pd_*z*_ after 6 h, followed by reuptake at 24 h (Figure S13). This observation suggests that normal cells possess a more robust exocytosis process for clearing Au_*x*_Cu_*y*_Pd_z,_ which may contribute to their greater tolerance of the material. Together with the results of the cellular uptake analysis, the substantial accumulation of Au_36_Cu_5_Pd_59_ micronanoshells within the cancer cells may help disrupt the balance of metal ion metabolism, ultimately leading to autophagy- and ferroptosis-induced cancer cell death (Figure 6e).

### Mechanisms of Au_36_Cu_5_Pd_59_-Induced Ferroptosis, Autophagy, and Cancer Cell Death

To determine whether dissolved Cu and Pd ions are responsible for the induction of cellular autophagy and apoptosis by the Au_36_Cu_5_Pd_59_ micronanoshells, we performed toxicity tests on both normal and cancer cells using equivalent concentrations of Cu and Pd ions as those in the micronanoshells. The results indicated that normal SV-HUC-1 cells strongly tolerate Cu and Pd metal ions (Figure S14a). Even at the equivalent concentration found in 0.5 mM Au_36_Cu_5_Pd_59_ micronanoshells, SV-HUC-1 cells maintained over 80% viability (Figure S14b). However, T24 cancer cells displayed significant toxicity at a concentration of 0.25 mM (Figure 7a), suggesting that cancer cells are relatively more sensitive to metal ions than normal cells are. Even though, the Au_36_Cu_5_Pd_59_ micronanoshells induced cellular toxicity at concentrations as low as 0.01 mM (Figure 3b), representing a difference of 1 order of magnitude (Figures 3b and 7a). In vitro release tests of Au_36_Cu_5_Pd_59_ micronanoshells revealed that about 65.7% of Cu ions were released at pH 7 and 75.2% at pH 4 after 24 h of aging, while less than 5% of Pd was detected (Figure S15). These results indicate that the concentration of free metal ions is significantly lower than the levels needed to cause cytotoxicity (Figure 7a). Therefore, we proposed that metal ions are not the main cause of the observed cellular autophagy and cell death, which are induced by the released copper ions (Figure S15) and the catalytic ROS activity^26,27^ (Figure 2g) of the remaining Pd domains (Figure S15) within the Au host material of Au_36_Cu_5_Pd_59_ nanomaterials.

**Figure 7:**
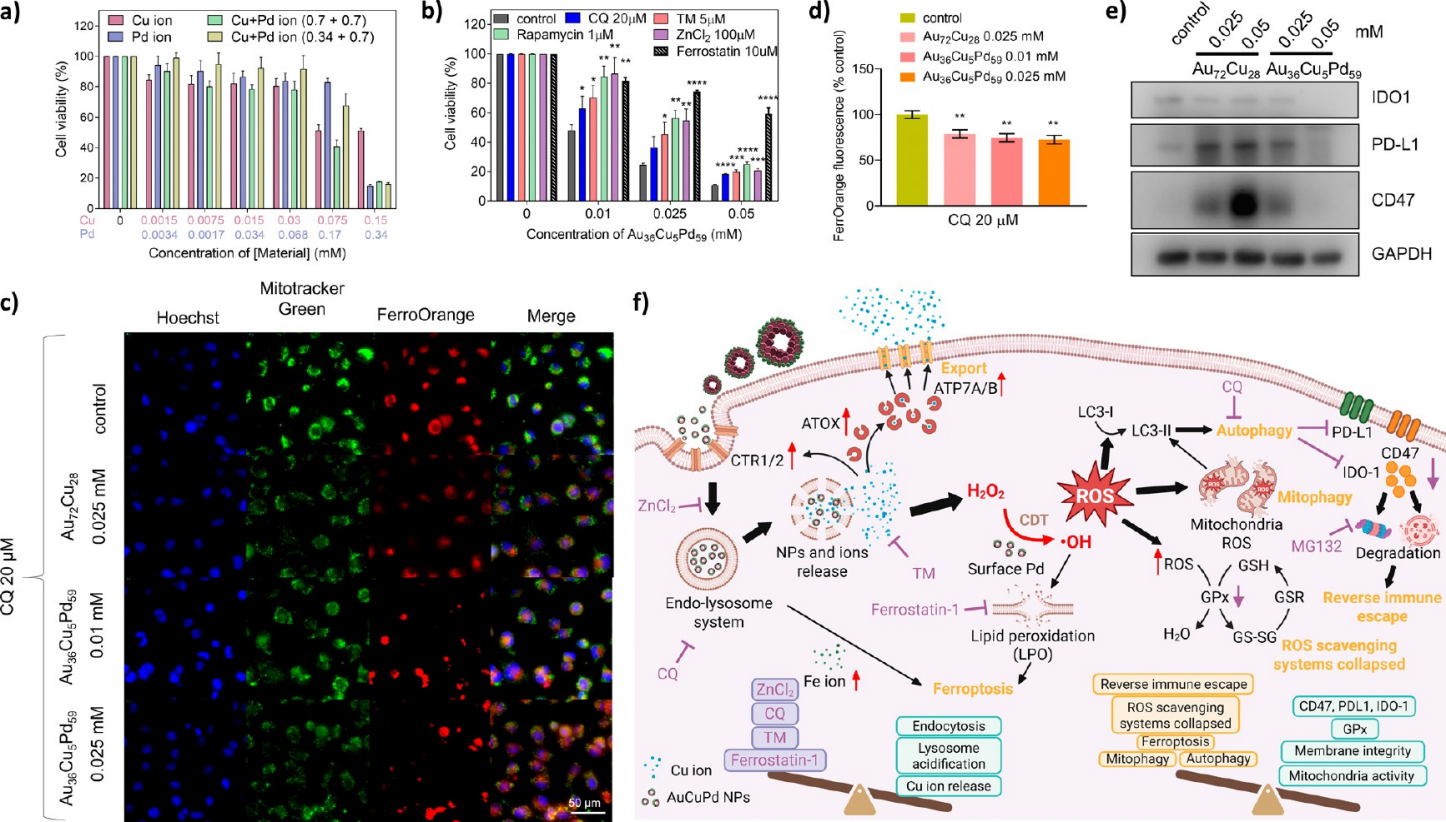
Cytotoxicity results of (a) MTT of T24 cells treated with equal amounts of metal ions for 24 h. (b) MTT results of Au_36_Cu_5_Pd_59_ micronanoshells combined with autophagy/Cu/ferroptosis inhibitors or activators. (c) Analysis of the intracellular labile iron level in Au_*x*_Cu_*y*_Pd_*z*_-treated T24 cells via FerroOrange staining under 20 μM CQ cotreatment for 16 h, and the fluorescence quantification results are presented in (d). Scale bar = 50 μm. (e) Changes in the expression levels of IDO1, PD-L1, and CD47 in T24 cells after treatment with indicated concentrations of Au_72_Cu_28_ or Au_36_Cu_5_Pd_59_ NP for 16 h, as determined by Western blotting. (f) The scheme illustrates that the Au_*x*_Cu_*y*_Pd_*z*_ NPs enhanced cancer cell uptake and induced ROS, which led to subsequent lipid peroxidation (ferroptosis), sustained autophagy (mitophagy), and Cu metabolism alterations (upregulated CTR1/2, ATOX, and ATP7A/B) and ultimately caused cell death. The sustained autophagy additionally triggered the degradation of immune evasion proteins (PD-L1, CD47, IDO-1) via lysosomes and proteasomes, resulting in the reversal of the immunosuppressive tumor microenvironment (right seesaw). Using inhibitors or reagents for blocking uptake, autophagy or copper chelation can significantly rescue cells from death.

Additional experiments were designed with inhibitors/inducers (Figure 7b) to elucidate the toxicity mechanism of Au_36_Cu_5_Pd_59_ micronanoshells and its association with cellular Cu uptake, autophagy, and exocytosis. Chloroquine (CQ) elevates lysosomal pH, thereby inhibiting lysosomal enzyme activity and subsequently blocking the autophagy pathway.^49^ The results revealed an increase in cell viability after being treated with CQ, suggesting that the toxicity of Au_36_Cu_5_Pd_59_ micronanoshells is related to the release of metal ions in the acidic lysosomal environment. Additionally, when we added the Cu chelator tetrathiomolybdate (TM)^50,51^ and ZnCl_2_ to compete for cellular Cu uptake,^52,53^ we found that they effectively reversed the cytotoxicity, indicating that the toxicity of Au_36_Cu_5_Pd_59_ micronanoshells may be increased by increasing Cu uptake through the CTR1 channel protein.^21^ By forcibly activating cellular exocytosis mechanisms with rapamycin,^54,55^ we observed a significant increase in cell viability. These findings suggest that enhancing the autophagy–exocytosis process can effectively facilitate the excretion of Au_36_Cu_5_Pd_59_ micronanoshells, reducing the toxicity of the material to cells. We also demonstrated inhibition of ferroptosis by ferrostatin-1,^56^ which acts as an antioxidant to remove lipid peroxides, can successfully reverse the cytotoxicity of Au_36_Cu_5_Pd_59_ (Figure 7c), indicating that ROS-mediated ferroptosis is also crucial for Au_36_Cu_5_Pd_59_-induced cell death. Additionally, we noted that the intracellular increase in labile iron induced by Au_36_Cu_5_Pd_59_ (Figure 5d,e) was diminished upon cotreatment with CQ (Figure 7c,d), suggesting that lysosomal degradation of Au_36_Cu_5_Pd_59_ is essential for initiating subsequent catalytic cascades.

Furthermore, T24 cells treated with Au_36_Cu_5_Pd_59_ micronanoshells presented decreased expression of the IDO1, PD-L1, and CD47 proteins (Figure 7e), which are known to facilitate immune evasion in cancer cells.^33^ Previous studies have indicated that autophagy may promote the degradation of PD-L1,^30,31^ thereby reducing the immune escapability of cancer cells. Using CQ (20 μM) and MG-132 (10 μM) to inhibit lysosomes^49^ and proteosomes^57^ can significantly restore the expression of IDO1, CD47, and PD-L1 (Figure S16a), suggesting that autophagy and proteosomes contribute to the degradation of these immune escape proteins (Figure 7f). Preliminary analysis revealed that Au_36_Cu_5_Pd_59_ treatment elevated global ubiquitination levels in total cell lysates (Figure S16b) and markedly increased the ubiquitination of immunoprecipitated PD-L1 (Figure S16c). These findings suggest a proteasome-mediated degradation pathway. Although this mechanism may extend to IDO1 and CD47, the detailed effects of the Cu/Pd ratios on these proteins remain under investigation for future research.

It should be noted that GPX4 was not restored when autophagy or the proteasome was blocked (Figure S16a), indicating that the decrease in GPX4 is due to ROS-mediated ferroptosis by Au_36_Cu_5_Pd_59_ micronanoshells (Figure 2g), rather than autophagy-mediated degradation. These findings suggest that the Au_36_Cu_5_Pd_59_ micronanoshells have the potential to reverse the immunosuppressive tumor microenvironment by inducing robust autophagy for protein degradation. Collectively, these results demonstrate that the Pd-mediated increase in ROS levels enhances cellular autophagy, modulates Cu uptake, and metabolism mechanisms, reverses the immunosuppressive status, and results in increased membrane oxidation, ultimately leading to cell death (Figure 7f).

Similar autophagy-linked cytotoxicity of Au_*x*_Cu_*y*_Pd_*z*_ NPs was also observed in the mouse bladder cancer cell line MB49. The results demonstrated that cell destruction (Figure S17) and autophagy (∼40%) significantly increased following 24 h of treatment with Au_36_Cu_5_Pd_59_ (Figure S18), whereas this increase was not observed in cells treated with Au_72_Cu_28._ Confocal imaging revealed that autolysosomes (Figure S19) were linked to increased mitochondrial ROS generation (Figure S20), elevated intracellular labile iron (Figure S21), and lipid peroxidation (Figure S22) due to the presence of Au_36_Cu_5_Pd_59_ micronanoshells, which occurred without significant apoptosis (Figure S23). Dark-field imaging revealed greater uptake of the Au_36_Cu_5_Pd_59_ micronanoshells than of the Au_72_Cu_28_ nanoshells (Figure S24). Inhibitor studies revealed that neutralizing the lysosomal pH or chelating intracellular Cu ions reversed the cytotoxicity of Au_36_Cu_5_Pd_59_ (Figure S25a) and reduced the increase in labile iron when cotreated with chloroquine (CQ) (Figure S25b). Metabolic pathway analysis revealed an increase in copper excretion, accompanied by no significant changes in the expression of copper uptake genes (Figure S25c). The disruption of cellular homeostasis and the promotion of cell death are enhanced by alterations in autophagy and copper uptake with Au_36_Cu_5_Pd_59_ micronanoshells, leading to increased membrane oxidation and, ultimately, cell death. This suggests that the use of Pd is a promising anticancer strategy.

We further validate the anticancer mechanisms of Au_*x*_Cu_*y*_Pd_*z*_ NPs using an orthotopic mouse bladder cancer model. This model enables simple monitoring of tumor growth and treatment effectiveness using ultrasound imaging. After the tumors were successfully established via ultrasound imaging, the tumors received four intravesical administrations of Au_*x*_Cu_*y*_Pd_*z*_ NPs twice a week, and tumor growth was monitored weekly (Figure 8a). The results showed that mice treated with 1 mM Au_36_Cu_5_Pd_59_ micronanoshells exhibited a 70% reduction in tumor size by Day 14 compared to the control groups (Figure 8b,c) and had a 1.5-fold increase in survival time, with a survival rate of up to 50% by Day 21. The control mice died on Day 14 (Figure 8d). The Au_86_Cu_14_ nanoshell was used for an in vivo comparison because it is a Pd-free composite and has an Au/Cu ratio of approximately 88:12, similar to that of the Au_36_Cu_5_Pd_59_ micronanoshells. Although Au_86_Cu_14_ nanoshells achieved 46% tumor growth inhibition, all mice died on Day 16, with only a slight increase in survival time (from 14 to 16 days). This suggests that the lack of Pd-mediated catalytic activity significantly decreased their anticancer effectiveness. The intravesical administration of Au_*x*_Cu_*y*_Pd_*z*_ NPs showed no significant toxicity, evidenced by stable body weight (Figure 8e) and no visible damage to major organs (Figure 8f). The biodistribution results (Figure 8g) showed that Au_36_Cu_5_Pd_59_ micronanoshells were only restricted to the bladder tumor site and were not detected in other organs. This result could be attributed to the local intravesical administration route, which can prevent potential systemic toxicity effects compared to the intravenous route.

**Figure 8:**
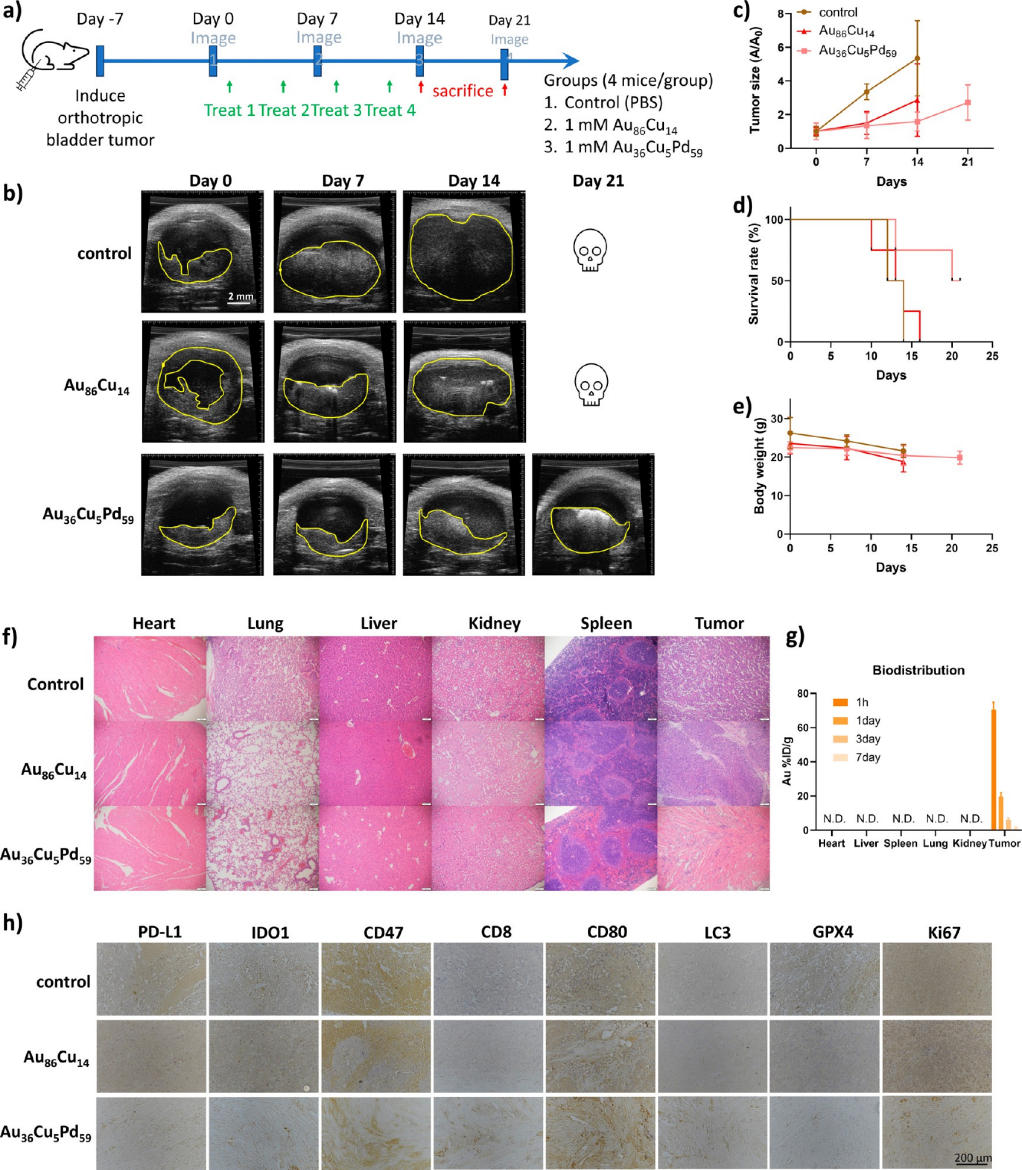
In vivo mechanism validation by orthotopic MB49 tumor-bearing mice. (a) Schematic illustration of treatment groups and experiment timelines for the mouse orthotopic bladder cancer model. Ultrasound imaging (b) and the normalized growth curve (c) of the tumor growth for the MB49 tumor-bearing mice received Au_36_Cu_5_Pd_59_ micronanoshells and Au_86_Cu_14_ nanoshells. The related survival rate and body weight curve are shown in (d,e), respectively. Scale bar = 2 mm in (b). (f) The major organ (heart, lung, liver, kidney, spleen, tumor) H&E staining images of Au_36_Cu_5_Pd_59_ micronanoshells, Au_86_Cu_14_ nanoshells, and particle-free groups. Scale bar = 100 μm. (g) The biodistribution of Au_36_Cu_5_Pd_59_ micronanoshells at different time points (1 h, 1 day, 3 days, and 7 days). N.D. means less than 0.1 ppm. (h) The tumor IHC images of Au_36_Cu_5_Pd_59_ micronanoshells, Au_86_Cu_14_ nanoshells, and particle-free groups for autophagy, ferroptosis, and immune cell markers. Scale bar = 200 μm.

We also investigated the occurrence of autophagy and ferroptosis in the tumors treated with Au_36_Cu_5_Pd_59_ micronanoshells using immunohistochemistry staining. As shown in Figure 8h, the tumors exhibited significantly elevated LC3 expression and reduced GPX4 levels, suggesting the induction of both autophagy and ferroptosis. Additionally, the decreased expression of Ki67 corroborated the growth inhibition findings from Figure 8b,c. The results also revealed a reduction in immunosuppressive markers such as PD-L1, CD47, and IDO1, along with an increase in the antitumor M1 macrophage marker CD80 and the cytotoxic T cell marker CD8. Such a reversed tumor’s immunosuppressive environment further validated the in vitro results presented in Figure 7e. Compared to Au_36_Cu_5_Pd_59_ micronanoshells, Au_86_Cu_14_ showed no significant changes in those markers except slightly reducing the GPX4 expression level, again confirming the importance of Pd incorporation for enhancing anticancer ability.

While Au_36_Cu_5_Pd_59_ micronanoshells can significantly inhibit tumor growth, we observed that tumor volume continued to increase after discontinuing its administration (Figure 8c). To address this, owing to the enhanced conversion of the photon energy to heat by Pd nanocrystals,^58,59^ we combined photothermal therapy (PTT) using Au_36_Cu_5_Pd_59_ micronanoshells—achieving an impressive photothermal conversion rate of up to 52.9% compared to no Pd counterpart (13.4%, Figure S26)—with 10 min of irradiation from an 808 nm laser (750 mW/cm^2^, Figure S27a). This approach with relatively low laser power density at 750 mW/cm^2^ and sample dose at 1 mM (600 μg/kg), as compared with the 1–1.5 W/cm^2^ and 2 mg/kg literature^60–62^ resulted in near-complete tumor eradication (Figure S27b,c) upon the treatment temperature around 45–46 °C of the MB49 tumor-bearing mice (Figure S28). Furthermore, immunohistochemical (IHC) examinations of the tumors revealed significant induction of autophagy and ferroptosis, along with an activated antitumor immune response in the Au_36_Cu_5_Pd_59_ micronanoshells with the PTT group (Figure S27d). These findings highlight that the autophagy–ferroptosis–immune activation pathway triggered by Au_36_Cu_5_Pd_59_ micronanoshells can serve as a powerful adjuvant mechanism, supporting other treatment modalities to achieve higher therapeutic efficiency or more complete tumor eradication.

Autophagy has also been shown to influence both ferroptosis and immune responses.^63–65^ Our in vitro (Figures 5c and 7e) and in vivo (Figure 8h) studies demonstrated that GPX4 protein levels and immunosuppressive markers decreased following treatment with Au_36_Cu_5_Pd_59_ micronanoshells, which was possibly associated with autophagy induction. This suggests that nanomaterial-induced autophagy may positively enhance ferroptosis and cancer immunity (Figure 7f), which warrants further research. Our preliminary in vivo results, combined with PTT, further suggest that integrating catheter-based light delivery could offer an effective strategy for in situ bladder cancer therapy in future clinical applications.

In recent years, researchers have shown increasing interest in the impact of Cu ions on cellular exocytosis^24,66^ and autophagy,^67,68^ yet the effects of nano-Cu on these processes remain unclear. Recently, leading international research teams have paid special attention to the biomedical applications of Cu-related nanomaterials, particularly in the context of the chemical dynamic mechanism for generating ROS inside cells, known as CDT.^45,46^ Compared with iron ions, Cu ions possess a greater capacity to decompose hydrogen peroxide, producing ROS.^69^ Cuprous ions exhibit a rate of ROS generation approximately 22 times higher than that of cupric ions.^70^ Most recently, Tsvetkov et al. demonstrated a new Cu-dependent form of cell death called cuproptosis, which is triggered by the targeting of Cu to mitochondria.^6^ Notably, the mitochondria were largely influenced by Au_36_Cu_5_Pd_59_ micronanoshells in T24 cells in the early periods of 0–4 h (Figure 4d). Considering the observed mitochondrial dysfunction, we cannot rule out the possibility that a trace amount of Cu in Au_*x*_Cu_*y*_Pd_*z*_ makes an additional contribution to the development of cuproptosis, which may be a future research target for killing T24 and MB49 cancer cells. Incorporating Pd into Au_*x*_Cu_*y*_Pd_*z*_ nanomaterials significantly boosts their catalytic activity, enabling robust intracellular ROS amplification and cascade reactions. This catalysis triggers enhanced cellular processes such as autophagy, mitochondrial damage, and lipid peroxidation, with additional immunomodulatory effects observed in both in vitro and in vivo studies. Importantly, Pd not only helps to overcome the immunosuppressive tumor microenvironment and improves therapeutic outcomes but also serves as an excellent photothermal agent for combined phototherapy strategies. The resulting autophagy-ferroptosis-immune reprogramming potential of Au_*x*_Cu_*y*_Pd_*z*_ suggests a promising avenue for enhancing the efficacy of immune checkpoint blockade by modulating the tumor microenvironment, thus offering improved treatment options for cancer patients.

## Conclusion

The ternary AuCuPd nanoalloy was successfully fabricated with a Cu nanotemplate, followed by a series of reactions of HAuCl_4_ and PdCl_2_. Our findings show that Pd-loaded Au_*x*_Cu_*y*_Pd_*z*_ micronanoshells enhance catalytic activity, increasing oxidative stress in cancer cells by converting hydrogen peroxide to hydroxyl radicals. The presence of Cu and Pd in the AuCuPd nanoalloy facilitated intracellular transport by binding to the Atox1 protein, which impedes autophagic flux. This induces autophagy and lipid peroxidation, alters Cu metabolism, and ultimately causes cell death. The high catalytic activity of the Au_36_Cu_5_Pd_59_ micronanoshells led to greater cellular uptake, resulting in ROS-related autophagy and lipid peroxidation that disrupted the redox balance and ultimately caused ferroptosis. Furthermore, Pd doping micronanoshells upregulated the expression of copper transporter genes (CTR1 and CTR2), enhancing nanoparticle uptake and elevating intracellular iron levels. Despite the increased expression of Atox1 and ATP7A/B, the accumulated nanoparticles remain unreleased, which hampers cell fate. Additionally, the Au_36_Cu_5_Pd_59_ micronanoshells help break down immune escape proteins, such as IDO1, PD-L1, and CD47, thereby reprogramming immunosuppression. These findings suggest that Au_36_Cu_5_Pd_59_-triggered autophagy may play a crucial role in ferroptosis and tumor immune regulation, providing insights into how multimetal NPs interact with both normal and cancer cells. Our preliminary in vivo studies, using PTT-free and PTT-intervened results, indicate that the metal composition and structure can significantly influence their biological effects when combined with other therapeutic strategies. Taken together, our findings offer valuable insights into designing multimetallic nanoparticles through element chemistry for cancer therapy, highlighting their potential to orchestrate autophagy-ferroptosis-immune-reprogramming pathways to boost therapeutic efficacy.

## Supplementary Material



## Notes

UV–visible absorption spectra, XPS spectra, TEM images, and AAS measurements of Au_*x*_Cu_*y*_Pd_*z*_; cell viability, cell fluorescence images, darkfield images, flow cytometry of SV-HUC-1 and MB49, and Western blot and gene expression analysis of MB49; in vivo tumor ultrasound images, tumor growth curve, H&E, and IHC stain; and primer sequences used in gene expression analysis (PDF)
