# Supplementary material for: Development of Au x Cu y Pd z Nanocomposites as Therapeutic Agents: Enhancing Cancer Treatment through Autophagy Modulation and Immune-Associated Effects
Source: ACS Appl Mater Interfaces. 2026 Mar 4;18(10):14540–57. doi: 10.1021/acsami.5c20536 (PMC13006959; doi:10.1021/acsami.5c20536)
Supplement: Supplementary file 1 [file am5c20536_si_001.pdf]

Supporting information

Development of $Au_xCu_yPd_z$ Nanocomposites as Therapeutic Agents: Enhancing Cancer Treatment through Autophagy Modulation and Immune-Associated Effects

Li-Xing Yang[1,2]†, Yi-Chun Chiu[3,4,5,6]†, Yi-Lun Chen[7], Ting-Ying Chen[7], Yi-Tseng Tsai[1,2], Yu-Cheng Chin[1,2], Ya-Ling Yeh[8], Ying-Jan Wang[8], Chih-Chia Huang[1,2,9]*, Rong-Jane Chen[8,10]* and Mei-Yi Liao[7]*

1. Department of Photonics, National Cheng Kung University, Tainan 701, Taiwan.
2. Center of Applied Nanomedicine and Core Facility Center, National Cheng Kung University, Tainan 701, Taiwan.
3. Division of Urology, Department of Surgery, Yangming Branch, Taipei City Hospital, Taipei 111, Taiwan.
4. Department of Urology, College of Medicine and Shu-Tien Urological Research Center, National Yang Ming Chiao Tung University, Taipei 112, Taiwan.
5. Department of Health and Welfare, University of Taipei, Taipei 111, Taiwan.
6. Department of Social and Public Affairs, University of Taipei, Taipei 111, Taiwan.
7. Department of Applied Chemistry, National Pingtung University, Pingtung 900, Taiwan.
8. Department of Environmental and Occupational Health, College of Medicine, National Cheng Kung University, Tainan 704, Taiwan.
9. Department of Medicinal and Applied Chemistry, Kaohsiung Medical University, Kaohsiung 807, Taiwan.
10. Department of Food Safety/Hygiene and Risk Management, College of Medicine, National Cheng Kung University, Tainan 704, Taiwan.

*Corresponding author

Prof. Chih-Chia Huang, Department of Photonics, National Cheng Kung University, No. 1, University Road, Tainan 701, Taiwan. Tel: +886-6-2757575 ext. 63913. E-mail: c2huang@mail.ncku.edu.tw

Prof. Rong-Jane Chen, Department of Food Safety/Hygiene and Risk Management, College of Medicine, National Cheng Kung University, Tainan 70428, Taiwan. Tel: 886-6-235-3535 Ext. 1542, Fax: 886-6-275-2484, Email: janekhc@gmail.com

Prof. Mei-Yi Liao, Department of Applied Chemistry, National Pingtung University, Pingtung 900, Taiwan. Tel: 886-8-766-3800 Ext. 33261, Fax: 886-8-7230305, E-mail: myliao@mail.nptu.edu.tw

†Li-Xing Yang and Yi-Chun Chiu contributed equally.

Table S1. Primer sequences for human genes.

Gene name		Sequence (5' → 3')
GAPDH	Forward primer	TGGTATCGTGGAAGGACTCATGAC
	Reverse primer	ATGCCAGTGAGCTTCCCGTTCAGC
ATP7A	Forward primer	GCCTCTGACACAAGAAACTG
	Reverse primer	GCTATTACTACCAACGGCTC
ATP7B	Forward primer	GCTGATTGGAAACCGTGAGT
	Reverse primer	ATTGCGATCATCCCACAGAG
CTR1	Forward primer	CCTTCTCACCATCACCCAAC
	Reverse primer	CTTGTGACTTACGCAGCAGG
CTR2	Forward primer	TCTTCTCAGATACAGCGGTGC
	Reverse primer	CTGATGGAGGTTGGCAGGTT
Atox1	Forward primer	ACTCTGCTTGCAACCCTGAA
	Reverse primer	CCTTTGGTCCATCCTGTGGG

Table S2. Primer sequences for mouse genes.

Gene name		Sequence (5' → 3')
GAPDH	Forward primer	TGGCATTGTGGAAGGGCTCATGAC
	Reverse primer	ATGCCAGTGAGCTTCCCGTTCAGC
ATP7A	Forward primer	GCCTCTGACCCAAGAAGCTG
	Reverse primer	GCTATCACTACCAGTGGCTC
ATP7B	Forward primer	TCCTCGCTGCACCCTATCT
	Reverse primer	GCGACTTTACAAGCCAGGGA
CTR1	Forward primer	GACAACATTACCATGCCACCTCACCA
	Reverse primer	GTAAAAACACTGCCACGAAGGCTCCA
CTR2	Forward primer	CTCTTTGATTTCTGGAGGGTCCACAG
	Reverse primer	TAGAATCCTGGTCTGGTCCCAAGATG
Atox1	Forward primer	GCAAACACCTTCGGCACAG
	Reverse primer	TCCAGCTTCCGTTCTGACTTG

Table S3. The DLS and zeta potential of $Au_xCu_yPd_z$ NPs.

	$Au_{72}Cu_{28}$	$Au_{69}Cu_{23}Pd_8$	$Au_{57}Cu_{16}Pd_{27}$	$Au_{36}Cu_5Pd_{59}$
DLS (nm)	48.6±24.8	57.7±29.2	62.5±31.3	77.9±38.2
PDI	0.261	0.256	0.251	0.240
Zeta potential (mV)	-56.5±7.4	-31.3±5.1	-18.7±4.1	-11.3±3.1

Table S4. The ratio of different oxidation states of Au, Cu and Pd in $Au_xCu_yPd_z$ NPs.

Au	Au^0	Au alloy	
$Au_{72}Cu_{28}$	4.9%	95.1%	
$Au_{69}Cu_{23}Pd_8$	21.0%	79.0%	
$Au_{57}Cu_{16}Pd_{27}$	41.3%	58.7%	
$Au_{36}Cu_5Pd_{59}$	40.8%	59.2%	
Cu	Cu^0	Cu^{2+}	Cu complex
$Au_{72}Cu_{28}$	48.3%	37.4%	14.3%
$Au_{69}Cu_{23}Pd_8$	73.1%	19.0%	7.9%
$Au_{57}Cu_{16}Pd_{27}$	65.7%	25.6%	8.7%
$Au_{36}Cu_5Pd_{59}$	62.6%	27.3%	10.2%
Pd	Pd^0	Pd^{2+}	Pd^{4+}
$Au_{72}Cu_{28}$	N/A	N/A	N/A
$Au_{69}Cu_{23}Pd_8$	65.7%	20.3%	14.0%
$Au_{57}Cu_{16}Pd_{27}$	63.7%	20.4%	15.9%
$Au_{36}Cu_5Pd_{59}$	58.1%	24.1%	17.8%

*The chemical states of Au,[1-2] Cu,[3-4] and Pd[5-6] atoms in the $Au_xCu_yPd_z$ nanostructures were assigned according to the literature.

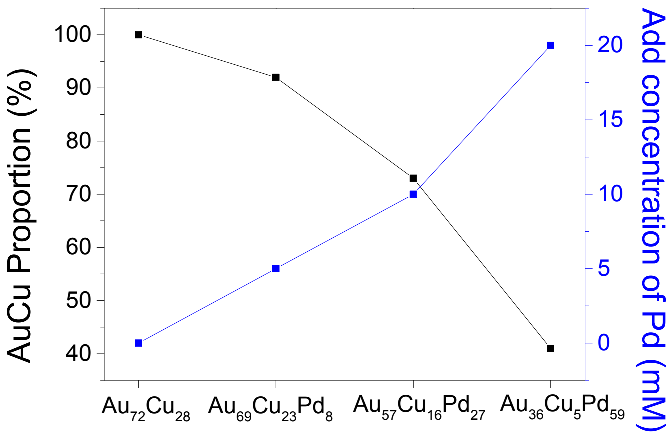

Figure S1. AAS measurements of the as-synthesized $Au_xCu_yPd_z$ NPs to determine the AuCu and Pd populations.

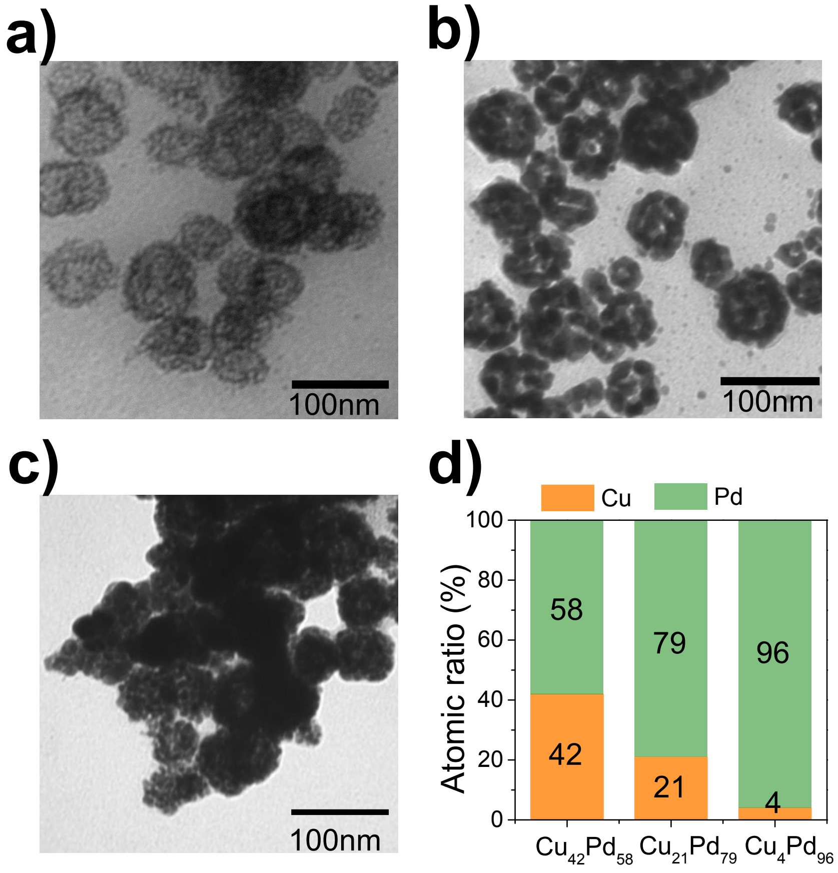

Figure S2. TEM images of the products from the reactions of Cu@PSMA NPs with Pd ions at (a) 5 mM, (b) 10 mM, and (c) 20 mM. AAS measurements for the results in a–c.

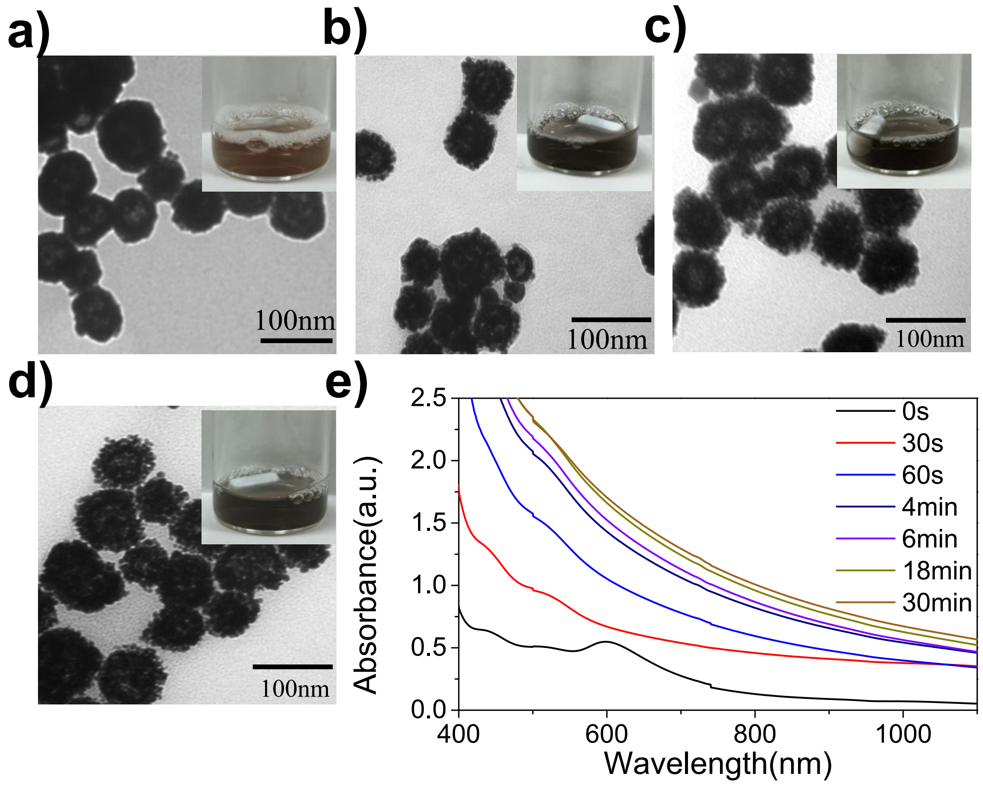

Figure S3. Time-dependent (a–d) TEM images and (e) UV–visible records for monitoring $Au_{36}Cu_5Pd_{59}$ NP generation at (a) 0 min (immediate), (b) 5 min, (c) 10 min, and (d) 20 min.

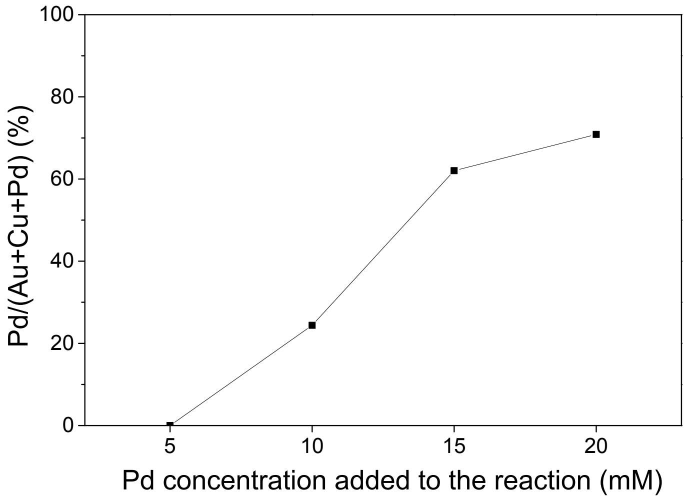

Figure S4. XPS analysis to determine the atomic ratio of Pd to the sum of Au, Cu, and Pd by adding different Pd concentrations

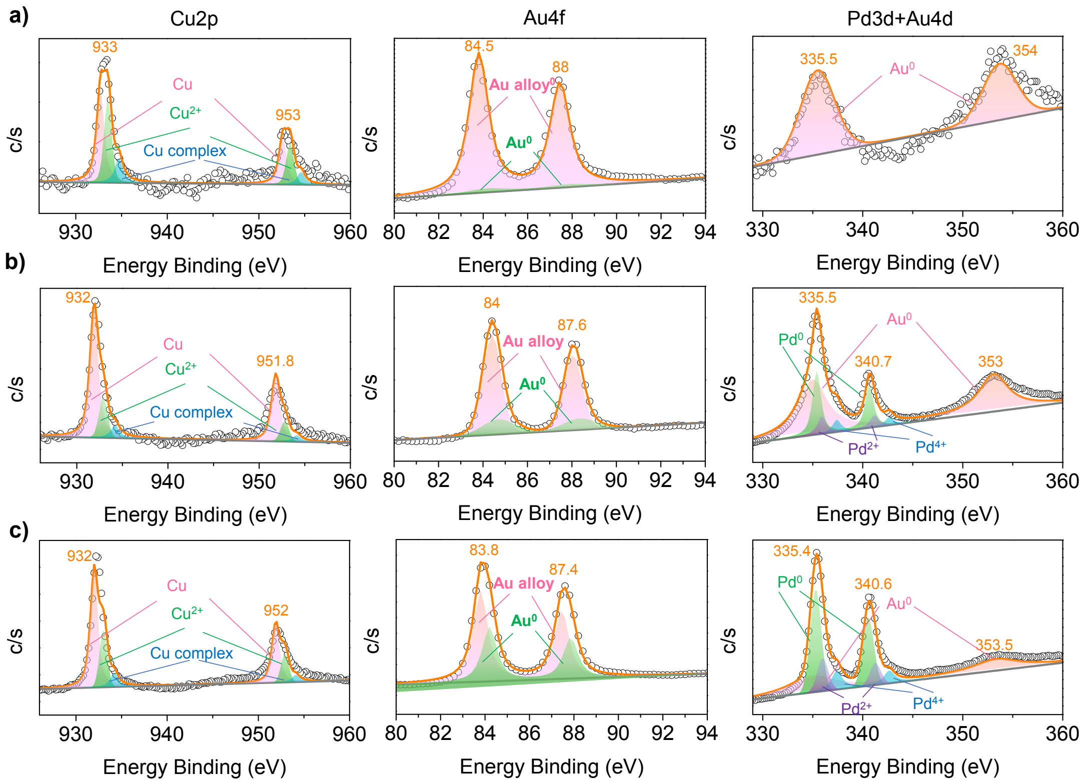

Figure S5. XPS spectra of the Cu 2p, Au 4f and Pd 3d+Au 4d core-level binding energies of (a) $Au_{72}Cu_{28}$, (b) $Au_{69}Cu_{23}Pd_{8}$, and (c) $Au_{57}Cu_{16}Pd_{27}$ nanostructures.

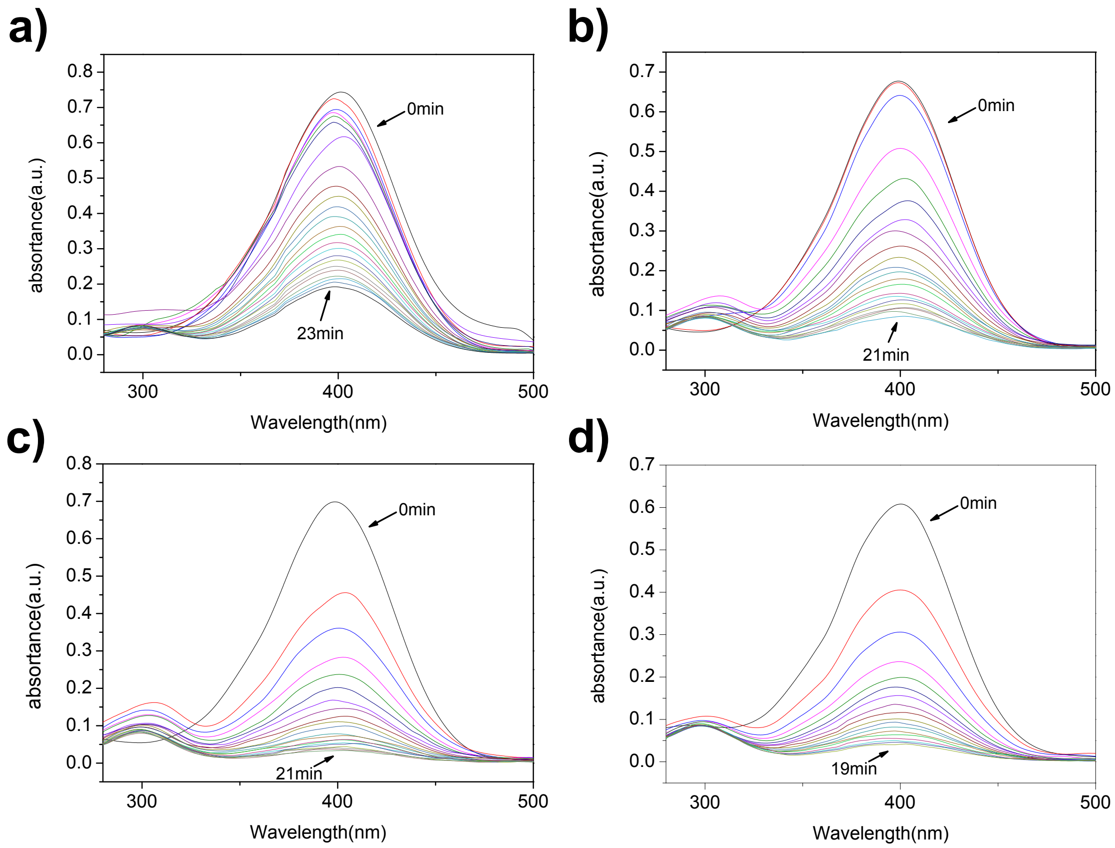

Figure S6. UV–vis spectra for monitoring the catalytic reaction of 4-NP with (a) $Au_{72}Cu_{28}$, (b) $Au_{69}Cu_{23}Pd_{8}$, (c) $Au_{57}Cu_{16}Pd_{27}$, and (d) $Au_{36}Cu_{5}Pd_{59}$ NPs.

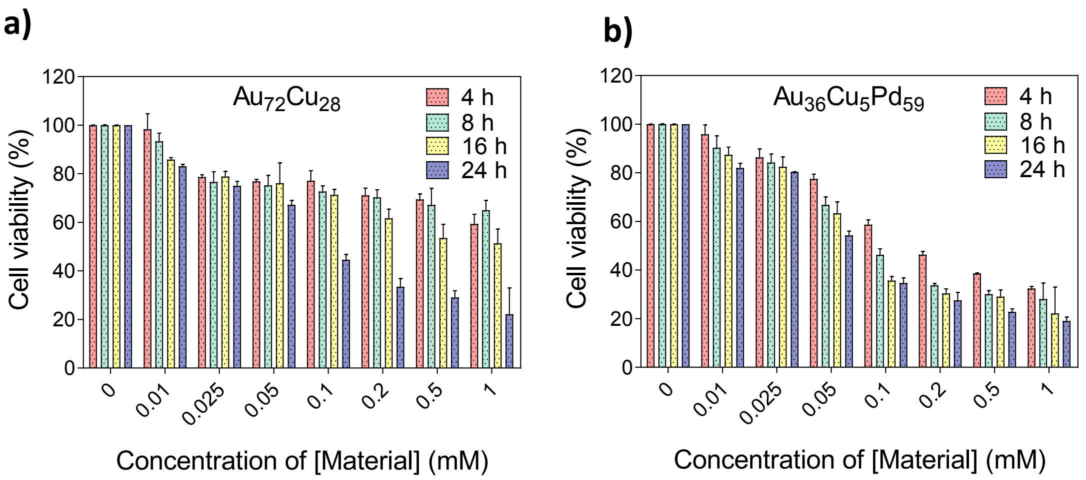

Figure S7. Cytotoxic effects of (a) $Au_{72}Cu_{28}$ and (b) $Au_{36}Cu_{5}Pd_{59}$ NPs on the SV-HUC-1 cell line (human ureteral epithelial cells) after different incubation times.

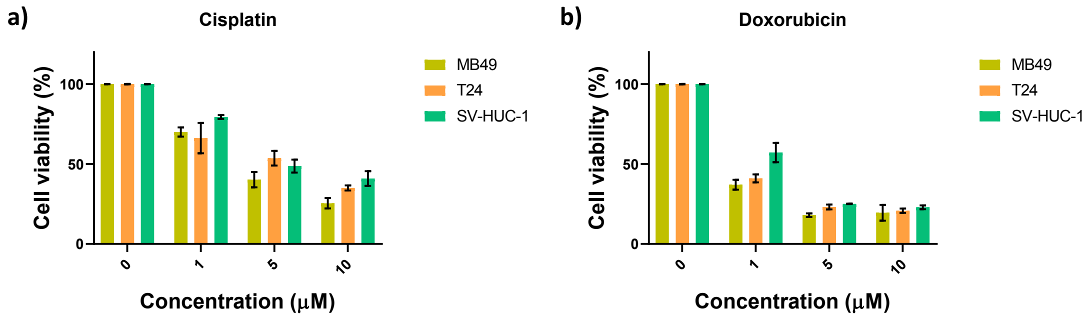

Figure S8. Cytotoxicity of (a) cisplatin and (b) doxorubicin to the MB29, T24 and SV-HUC-1 cell lines at different concentrations after 24 h of incubation.

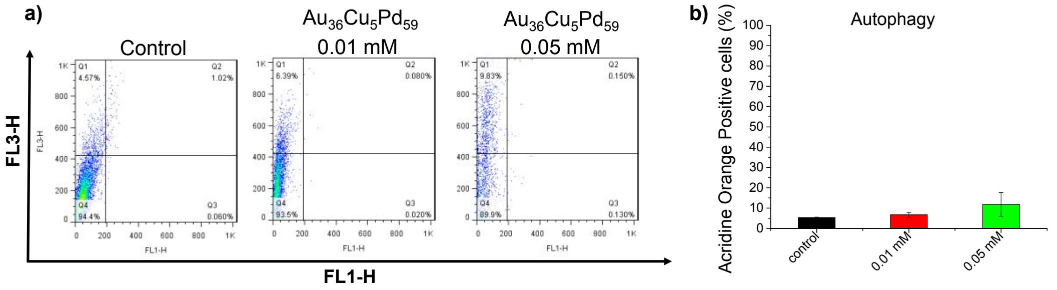

Figure S9. (a) Flow cytometry analysis of autophagy induction in SV-HUC-1 cells (10,000 cells counted) treated with different concentrations of $Au_{36}Cu_5Pd_{59}$. The results are statistically analyzed with three replicates, as shown in (b).

To achieve a more comprehensive comparison group in the biological examination, we also fabricated $Au_{90}Cu_{10}$ nanoshells (Figure S10a), which have a similar Au/Cu ratio to $Au_{36}Cu5Pd_{59}$, as reported previously. [3] It showed an absorption spectrum with λ_{max} at 583 nm (Figure S10b) due to the SPR feature. After co-culturing with T24 cells, the biological responses of these $Au_{86}Cu_{14}$ nanoshells were consistent with the cell viability and autophagy results of the $Au_{72}Cu_{28}$ group in Figure 3 in the main text, showing no significant cytotoxicity (Figure 10c) or autophagy induction, as measured by flow cytometry (Figure 10d), immunofluorescence staining (Figure S10e) and western blotting (Figure S10f). These findings suggest that Pd in $Au_{36}Cu_5Pd_{59}$ micro-nanoshells primarily drives cytotoxicity and autophagy induction in cancer cells.

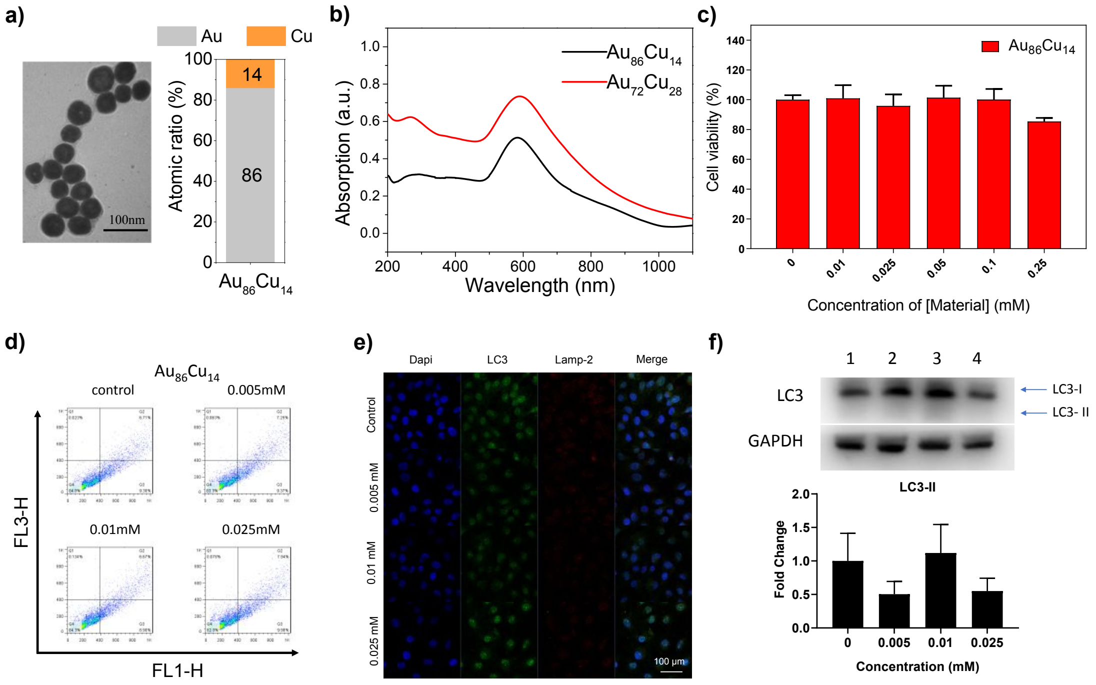

Figure S10. TEM image, AAS analysis (a) and UV-Visible spectrum (b) of $Au_{86}Cu_{14}$ nanostructures. (c) MTT results of $Au_{86}Cu_{14}$ nanostructures incubation with T24 cells for 24 hours. (d) The autophagy analysis of $Au_{86}Cu_{14}$ in T24 cells by AO staining and measured via flow cytometry. (e) Confocal microscopy images of T24 cells after treatment with different concentrations of $Au_{86}Cu_{14}$ nanostructures for 24 h, followed by LC3 and Lamp-2 immunofluorescence staining. Scale bar = 100 μm. (f) Changes in the expression levels of the autophagy protein LC3-II in T24 cells after treatment with different concentrations of $Au_{86}Cu_{14}$ nanostructures for 24 h, along with the quantification results and concentration order.

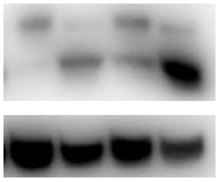

Figure S11. Changes in the expression levels of the autophagy protein LC3-II in T24 cells after treatment with CQ and $Au_{36}Cu_5Pd_{59}$ nanostructures for 16 h

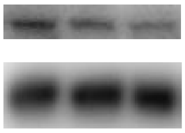

Figure S12. Changes in the expression levels of NRF2 in T24 cells after treatment with different concentrations of $Au_{36}Cu_5Pd_{59}$ micro-nanoshells for 16 h.

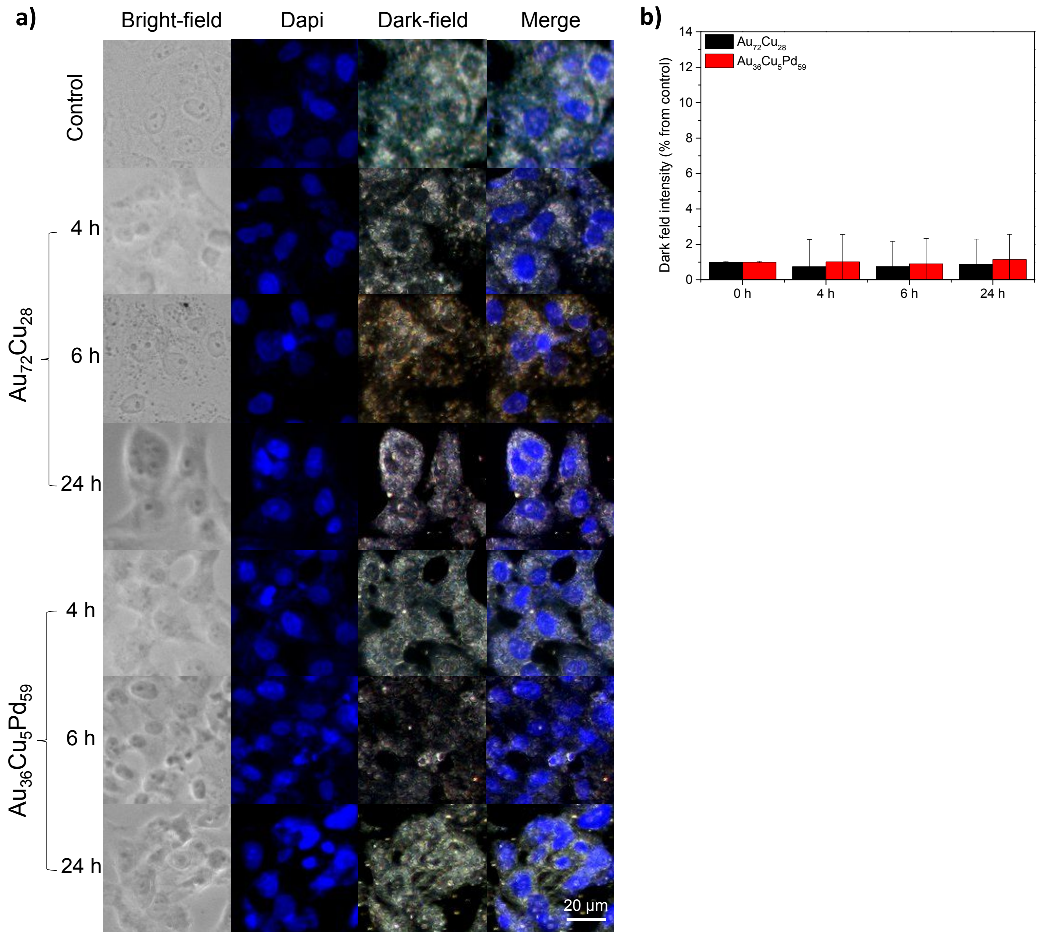

Figure S13. (a) Dark-field microscopy images of SV-HUC-1 cells treated with 0.025 mM $Au_{72}Cu_{28}$ and $Au_{36}Cu_5Pd_{59}$ NPs at different time points (0–24 h). (b) Normalized intensity of intracellular dots from the darkfield images in (a). Scale bar = 20 µm.

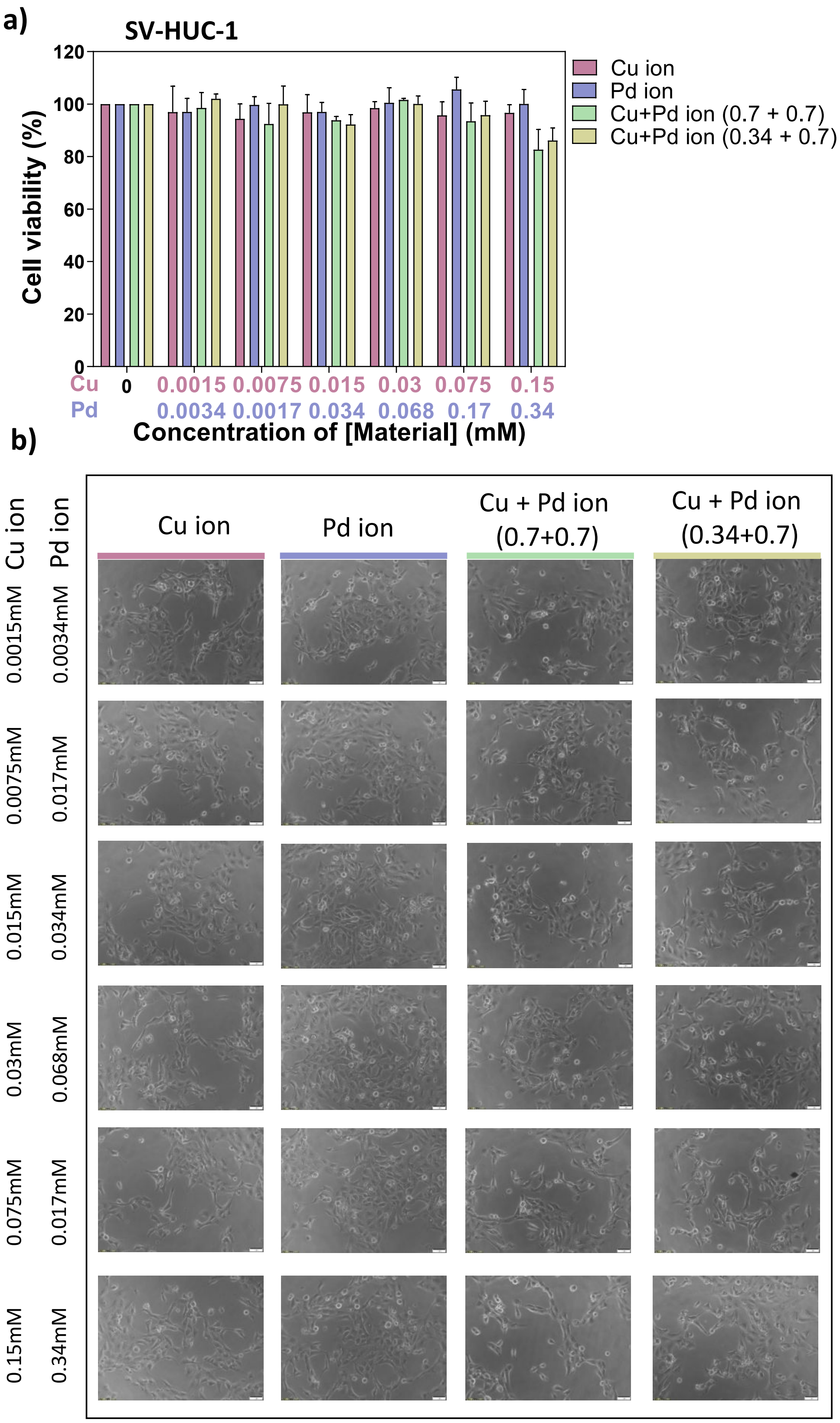

Figure S14. The cytotoxicity results of SV-HUC-1 cells treated with an equal amount of metal ions: (a) MTT assay for cell viability and (b) bright-field images of the cells. Scale bar = 20 µm.

The Cu release results indicated that around 65.7% of Cu was released within the physiological environment (4-24 hours), rising slightly to 78.7% after 7 days. When these micro-nanoshells were dispersed in acidic PBS buffer, Cu concentration increased by an additional 9.5-10.7%. Less than 5% of Pd was detected over the 7-day aging period (see Figure S15), suggesting that the released metal ions were significantly below the levels needed to cause ion-induced cytotoxicity.

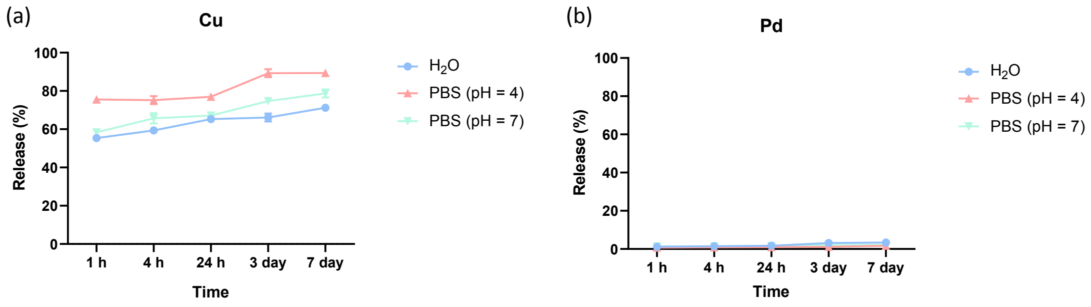

Figure S15. The ion release profiles of Cu and Pd from the $Au_{36}Cu_5Pd_{59}$ micro-nanoshells in different buffer conditions.

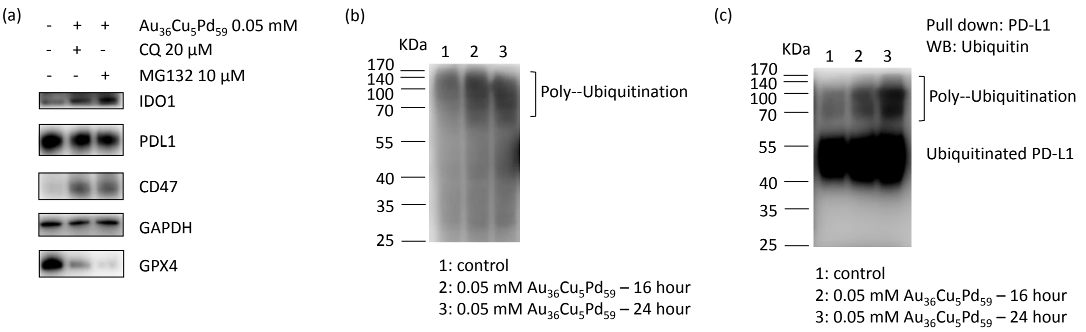

Figure S16. (a) Changes in the expression levels of the IDO1, CD47, PD-L1, and GPX4 in T24 cells after treated with $Au_{36}Cu_5Pd_{59}$ and CQ (20 μM) or MG-132 (10 μM) for 24 h. Changes in the expression levels of ubiquitin in (b) total cell lysate and (c) immunoprecipitated PD-L1 after treated with 0.05 mM $Au_{36}Cu_5Pd_{59}$ for 16 and 24 h.

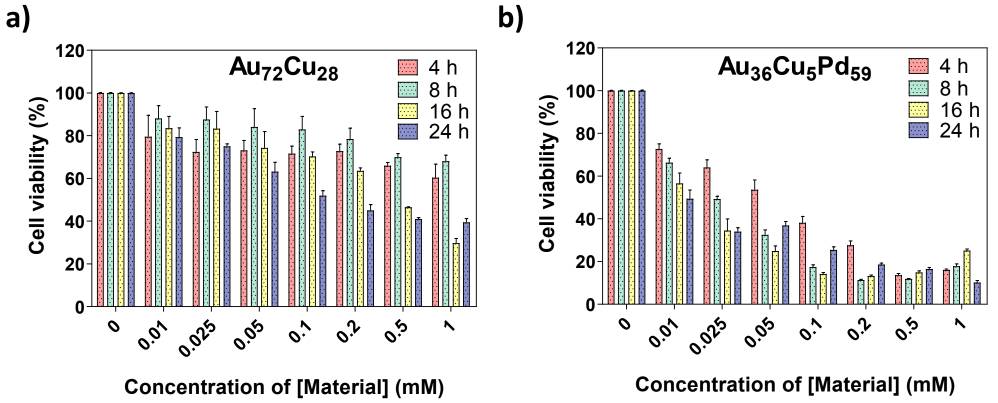

Figure S17. Cytotoxicity of (a) the $Au_{72}Cu_{28}$ and (b) the $Au_{36}Cu_5Pd_{59}$ NPs to the MB49 cell line (a mouse bladder cancer cell line) after different treatment times.

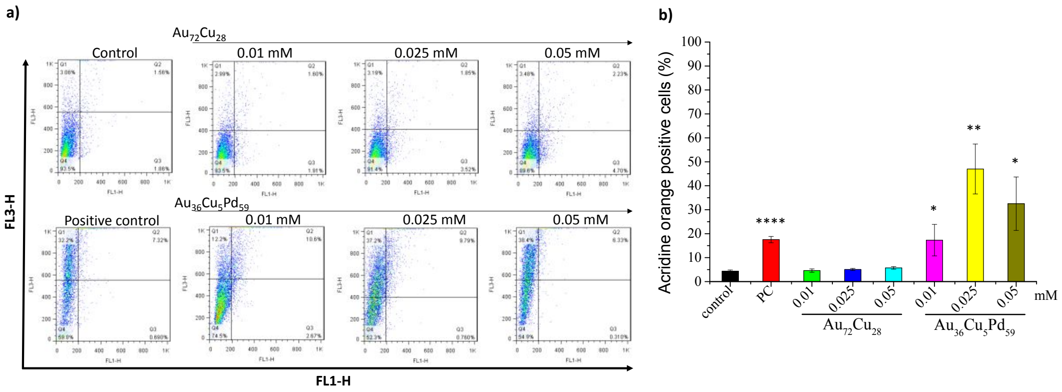

Figure S18. (a) Flow cytometry analysis of autophagy induction in MB49 cells treated with different concentrations of the $Au_{72}Cu_{28}$ and $Au_{36}Cu_5Pd_{59}$ NPs for 24 h. The results were statistically analyzed, with three replicates shown in (b).

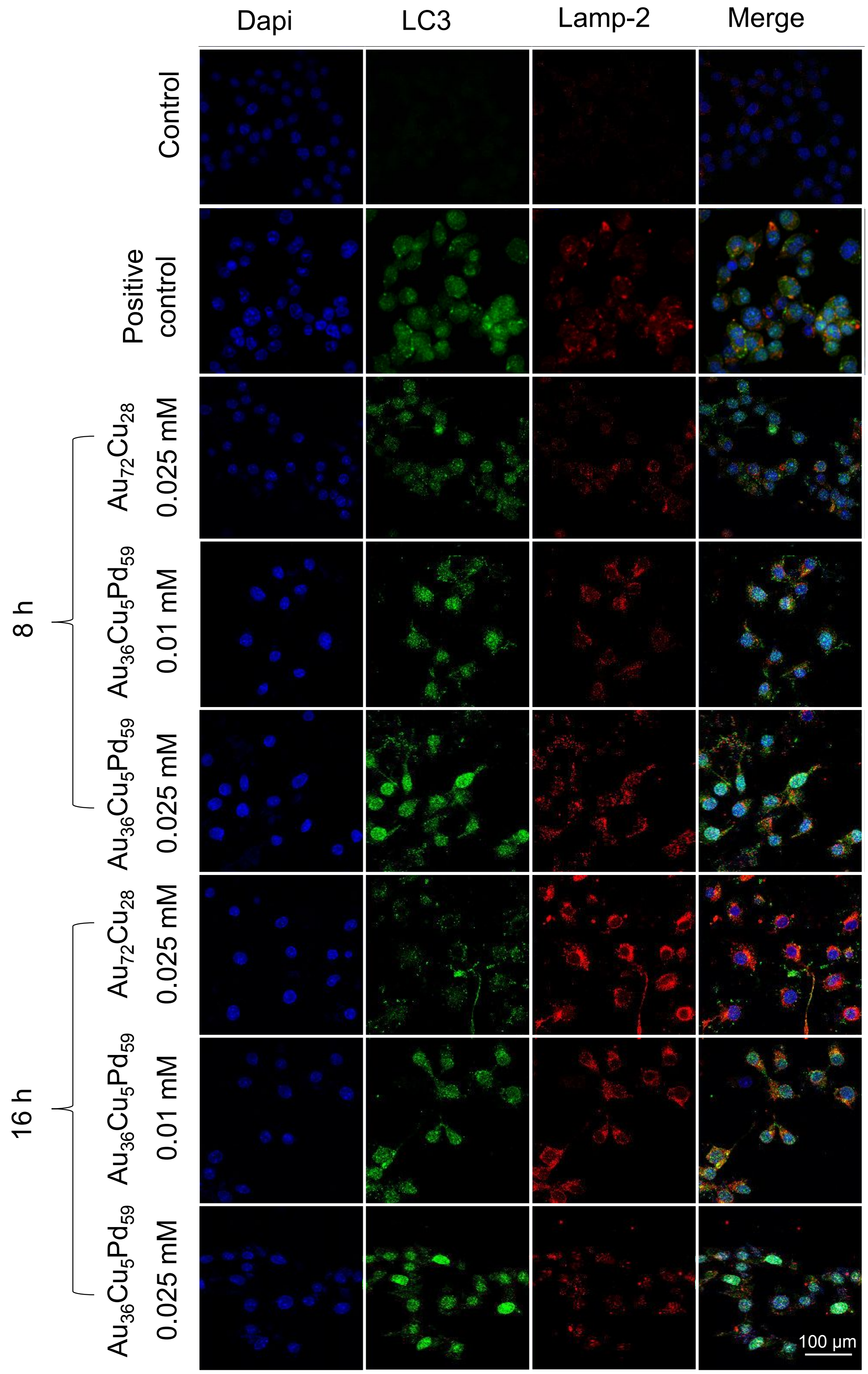

Figure S19. Confocal microscopy images of MB49 cells after treatment with different concentrations of $Au_{72}Cu_{28}$ and $Au_{36}Cu_5Pd_{59}$ for 24 h, followed by LC3 and Lamp-2 immunofluorescence staining. Scale bar = 100 μm.

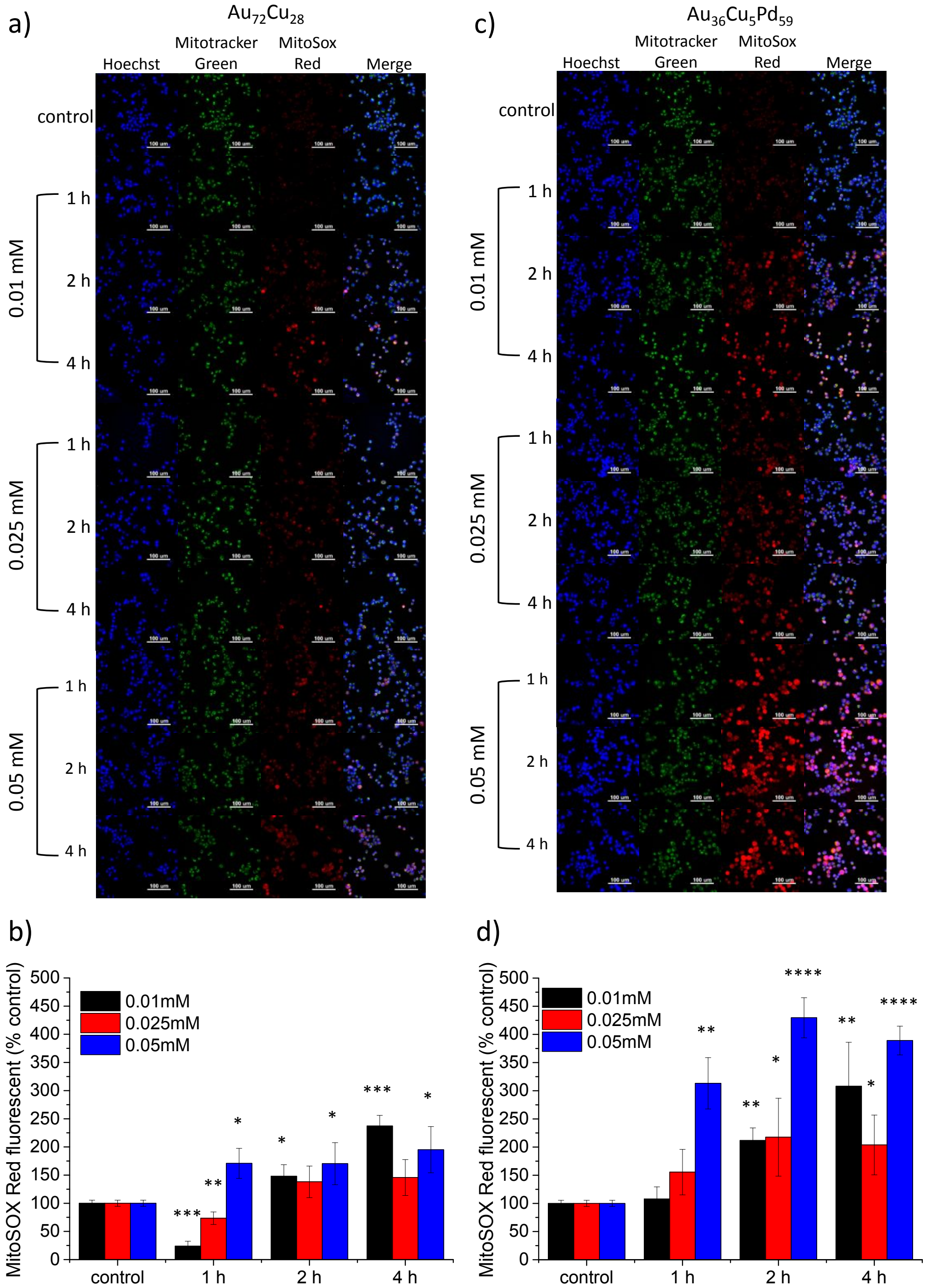

Figure S20. Fluorescence images of mitochondrial ROS in MB49 cancer cells treated with different concentrations of (a) $Au_{72}Cu_{28}$ and (c) $Au_{36}Cu_5Pd_{59}$ after 1, 2 and 4 h of culture. The respective quantitation results are presented in (b) and (d). Scale bar = 100 μm.

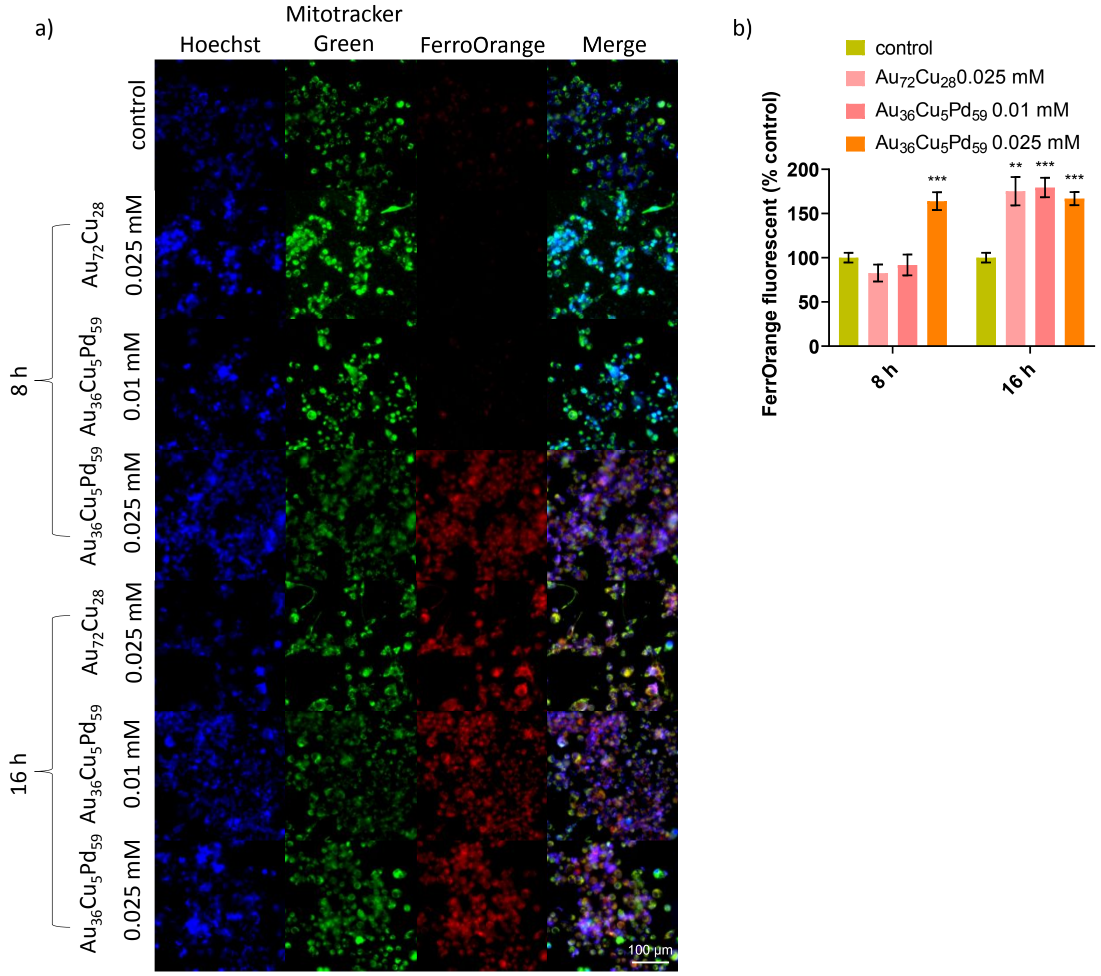

Figure S21. (a) Intracellular labile iron level analysis by FerroOrange staining in $Au_xCu_yPd_z$ NP-treated MB49 cells. The quantification results are presented in (b). Scale bar = 100 μm.

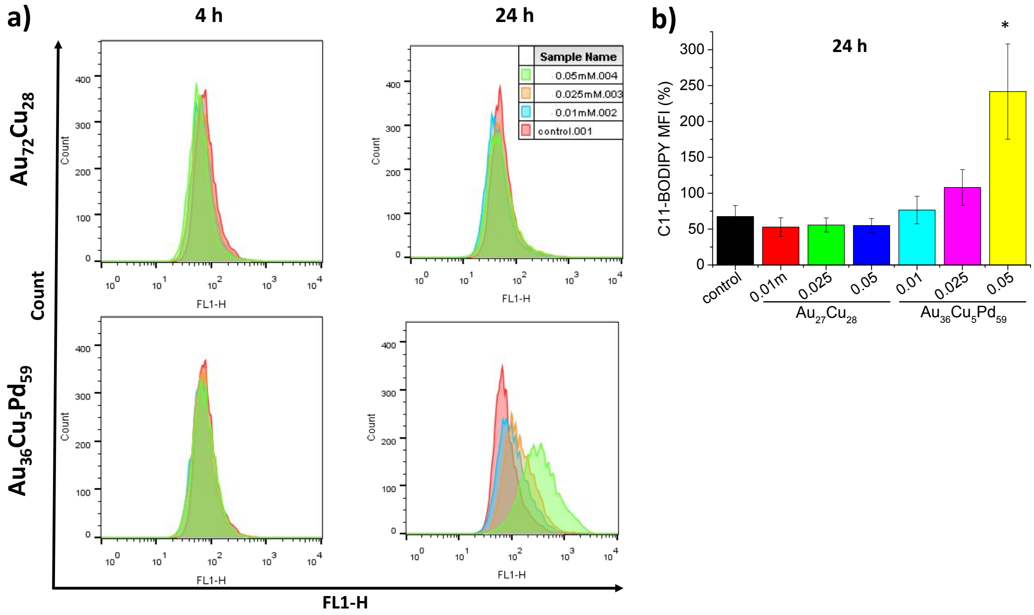

Figure S22. (a) Results of lipid peroxidation in MB49 cancer cells treated with different concentrations of $Au_{72}Cu_{28}$ and $Au_{36}Cu_5Pd_{59}$ after 4 and 24 h of incubation. (b) Statistical analysis of LPO ratios from triplicate experiments in (a).

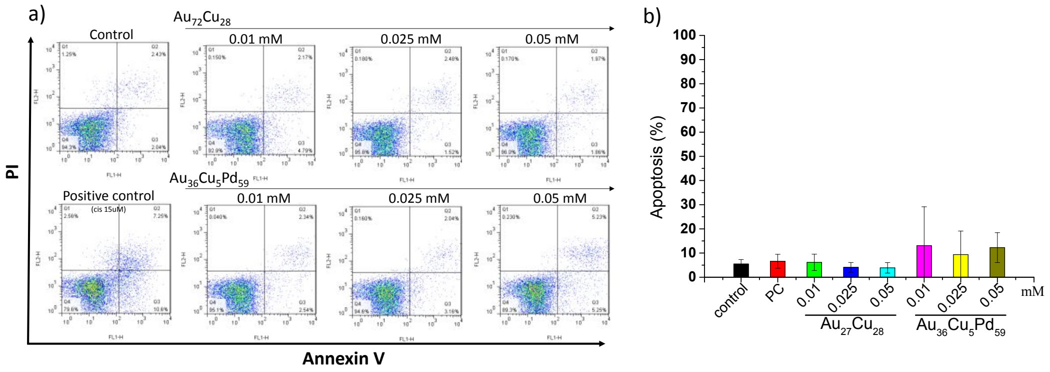

Figure S23. The apoptosis results of cancer cells treated with different concentrations of $Au_{72}Cu_{28}$ and $Au_{36}Cu_5Pd_{59}$ via flow cytometric analysis of MB49 cells after 24 h of incubation. (b) Statistical analysis of apoptosis ratios from triplicate experiments in (a). There is no statistic significance between control and treatment groups.

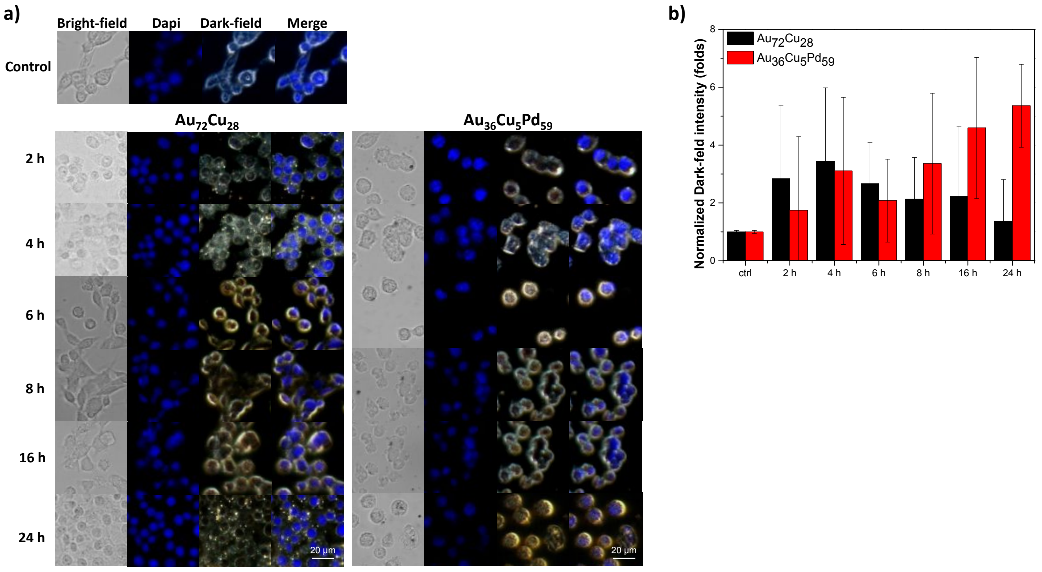

Figure S24. (a) Dark-field microscopy images of MB49 cells treated with 0.025 mM $Au_{72}Cu_{28}$ or $Au_{36}Cu_5Pd_{59}$ NPs at different time points (0–24 h). (b) Normalized intensity of intracellular dots from the darkfield images in (a). Scale bar = 20 μm.

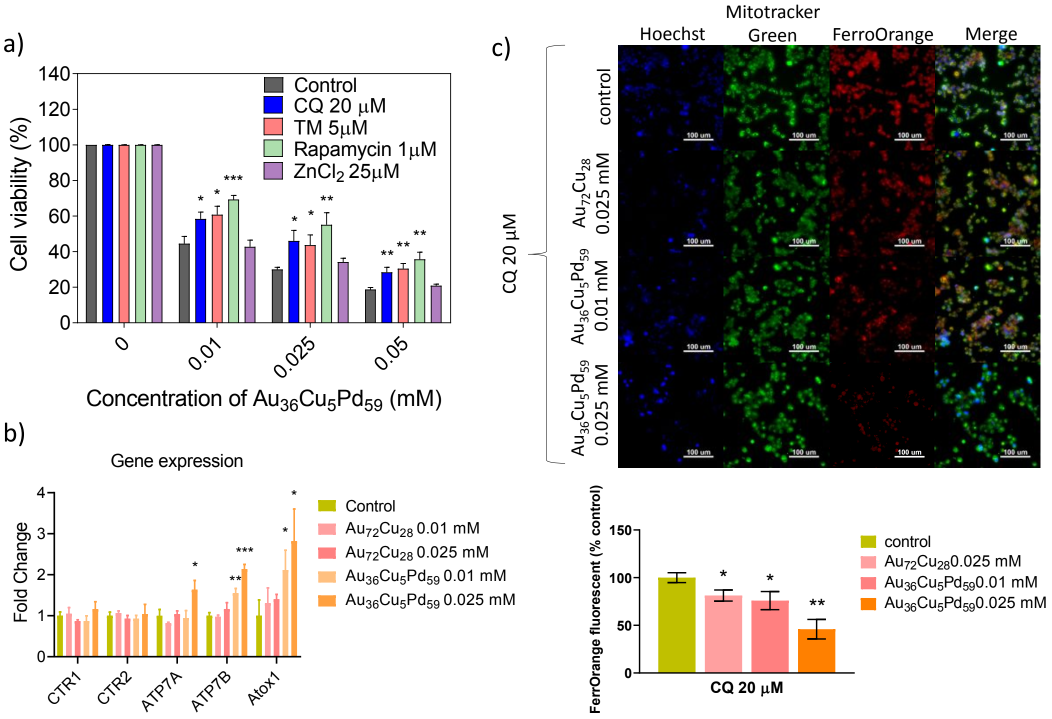

Figure S25. (a) Cytotoxicity results of MB49 cells treated with $Au_{36}Cu_5Pd_{59}$ combined with autophagy/copper inhibitors or activators. (b) Copper metabolism-related gene expression of MB49 treated with the $Au_{72}Cu_{28}$ and $Au_{36}Cu_5Pd_{59}$ NPs for 24 h. (c) Intracellular labile iron level analysis by the red fluorescence of FerroOrange staining under 20 μM CQ cotreatment in MB49 cells and quantification of the results. Scale bar = 100 μm.

In vitro photothermal performance of $Au_xCu_yPd_z$ and photothermal conversion efficiency (η) calculation

0.2 mL of $Au_xCu_yPd_z$ solution with different concentration (0.1, 0.3, 1 mM) was added into 96-well transparent plate, followed by irradiating via an 808 nm laser light with 0.5, 0.75 or 1 W/cm^2 power density for 10 min. The solution temperature increment was measured by the t-type thermocouple thermometer with a thermocouple wire to determine the NIR response photothermal conversion.

The photothermal conversion efficiency (η) could be calculated by the formula described by Roper et al.[7]

$$\eta = \frac{hS\Delta T_{max} - Q}{I(1-10^{-A})} \qquad \text{Equation 1}$$

In the formula, h is indicated to the heat transfer coefficient and S is assigned to the surface area of the container. Water photothermal effect (Q) was measured as 0.00224 mW by an independent experiment. ΔTmax is the temperature differential value between maximal steady-state temperature and the outset, which is 34.8 for $Au_{36}Cu_5Pd_{59}$ and 11.0 for $Au_{86}Cu_{14}$. Qs is the heat held by the NIR light absorbance of the surrounding liquid. I is the laser power which is 1 W/cm^2. Lastly, A is the absorbance of the nanoparticles at 808 nm.

$$\theta = \frac{T - T_{sur}}{T_{max} - T_{sur}} \qquad \text{Equation 2}$$

$$\tau = \frac{\sum M_i C_i}{hS} \qquad \text{Equation 3}$$

The value of hS is derived from equation 3 below, where τ is the sample system time constant which showed in Figure S23a and S23d and calculated the slope of linear fitting trend line by –ln(1-θ) versus time (Figure S23c and S23e). Mi and Ci are the mass and the heat capacity of the solution and container.

For $Au_{36}Cu_5Pd_{59}$

138.83 = [(0.2 + 0.38 × 1.34) × 4.2]/hS, hS = 0.0215

$\eta = (0.0215 \times 34.8 - 0.00224)/[1(1 - 10^{-0.538}))$ = 52.9%

For $Au_{86}Cu_{14}$

200.16 = [(0.2 + 0.38 × 1.34) × 4.2]/hS, hS = 0.0149

$\eta = (0.0149 \times 11 - 0.00224)/[1(1 - 10^{-0.408}))$ = 13.4%

By calculating the equation, the photothermal conversion efficiency value of $Au_{36}Cu_5Pd_{59}$ is 52.9%, which is way beyond the one of $Au_{86}Cu_{14}$ (13.4%). The enhancement of photothermal conversion efficiency was assigned to the surface plasmon resonance (SPR) of Pd[8-9] to improve the absorption at NIR region.

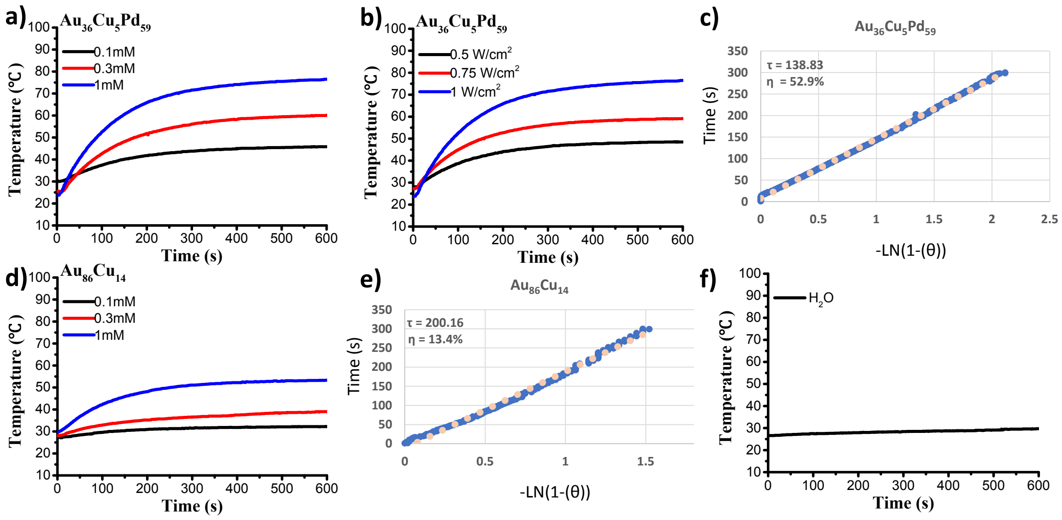

Figure S26. (a) NIR photothermal curves of $Au_{36}Cu_5Pd_{59}$ at various concentrations (0.1, 0.3, and 1 mM) under the laser irradiation of NIR (808 nm, 1.0 W/cm^2) for 10 min. (b) Photothermal curves of $Au_{36}Cu_5Pd_{59}$ (1 mM) at various 808 laser power densities (0.5, 0.75, and 1 W/cm^2). (c) Calculation of the photothermal conversion efficiency (η) of $Au_{36}Cu_5Pd_{59}$ at 808 nm. (d) NIR photothermal curves of $Au_{86}Cu_{14}$ at various concentrations (0.1, 0.3, and 1 mM) under the laser irradiation of NIR (808 nm, 1.0 W /cm^2) for 10 min. (e) Calculation of the photothermal conversion efficiency (η) of $Au_{86}Cu_{14}$ at 808 nm. (f) NIR photothermal curves of water at 1.0 W/cm^2 808 nm laser power densities.

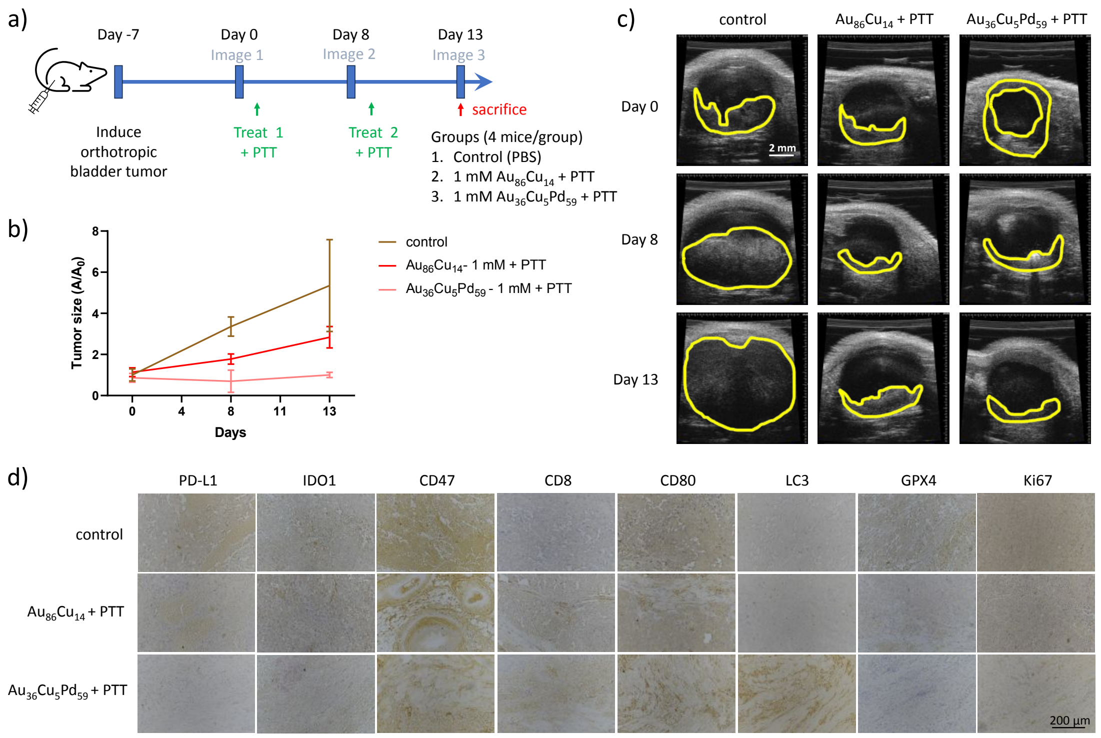

Figure S27. *In vivo* mechanism validation of PTT combined therapeutic effects by orthotropic MB49 tumor-bearing mice. (a) Schematic illustration of treatment groups and experiment timelines for the mouse orthotropic bladder cancer model with PTT therapy (808 nm laser with 750 mW/cm^2 for 10 min). Ultrasound imaging (b) and the normalized growth curve (c) of the tumor growth for the MB49 tumor-bearing mice received $Au_{36}Cu_5Pd_{59}$ micro-nanoshells and $Au_{86}Cu_{14}$ nanoshells plus light irradiation. (d) The tumor IHC images of $Au_{36}Cu_5Pd_{59}$ micro-nanoshells, $Au_{86}Cu_{14}$ nanoshells, and particle-free groups (with light irradiation) for autophagy, ferroptosis, and immune cell markers. Scale bar = 200 µm.

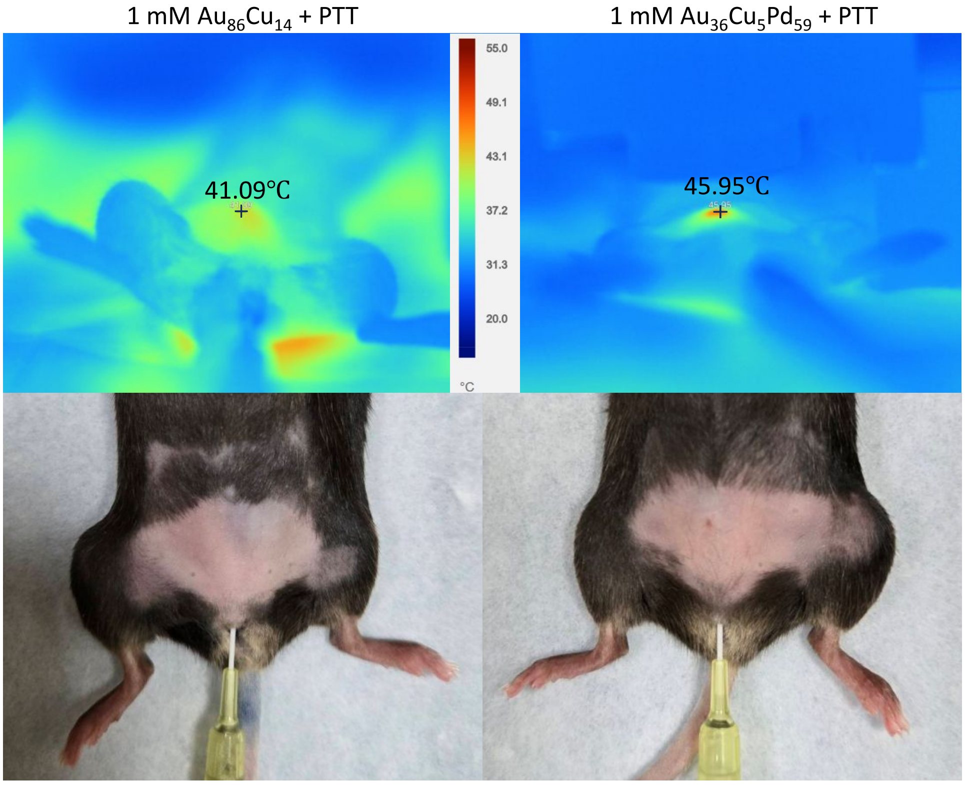

Figure S28. Photographic images of $Au_{86}Cu_{14}$ and $Au_{36}Cu_{5}Pd_{59}$ micro-nanoshells treated mouse, followed by recording with a thermograph camera to detect the local heat under 808 nm laser irradiation at 0.75 W/cm^2.

Reference

1. Huang, T. C.; Tsai, H. C.; Chin, Y. C.; Huang, W. S.; Chiu, Y. C.; Hsu, T. C.; Chia, Z. C.; Hung, T. C.; Huang, C. C.; Hsieh, Y. T., Concave Double-Walled AgAuPd Nanocubes for Surface-Enhanced Raman Spectroscopy Detection and Catalysis Applications. *Acs Appl Nano Mater* **2021,** *4* (10), 10103-10115.

2. Hepperle, P.; Herman, A.; Khanbabaee, B.; Baek, W. Y.; Nettelbeck, H.; Rabus, H., XPS Examination of the Chemical Composition of PEGMUA-Coated Gold Nanoparticles. *Part Part Syst Char* **2022,** *39* (9), 2200070.

3. Chuang, Y. T.; Cheng, T. Y.; Kao, T. L.; Liao, M. Y., Hollow AuxCu1-x Alloy Nanoshells for Surface-Enhanced Raman-Based Tracking of Bladder Cancer Cells Followed by Triggerable Secretion Removal. *Acs Appl Nano Mater* **2020,** *3* (8), 7888-7898.

4. Liarou, E.; Han, Y. S.; Sanchez, A. M.; Walker, M.; Haddleton, D. M., Rapidly self-deoxygenating controlled radical polymerization in water disproportionation of Cu(i). *Chemical Science* **2020,** *11* (20), 5257-5266.

5. Wang, J.; Shi, J.; Yin, K.; Meng, F.; Wang, S.; Lou, L.; Zhou, J.; Xu, X.; Wu, H.; Luo, Y.; Li, D.; Chen, S.; Meng, Q., Pd(II)/Pd(IV) redox shuttle to suppress vacancy defects at grain boundaries for efficient kesterite solar cells. *Nat Commun* **2024,** *15* (1), 4344.

6. Chetyrin, I. A.; Bukhtiyarov, A. V.; Prosvirin, I. P.; Khudorozhkov, A. K.; Bukhtiyarov, V. I., In Situ XPS and MS Study of Methane Oxidation on the Pd-Pt/Al_2O_3. Catalysts. *Top Catal* **2020,** *63* (1-2), 66-74.

7. Roper, D. K.; Ahn, W.; Hoepfner, M., Microscale Heat Transfer Transduced by Surface Plasmon Resonant Gold Nanoparticles. *J Phys Chem C Nanomater Interfaces* **2007,** *111* (9), 3636-3641.

8. Wang, W.; Chen, C.; Ying, Y.; Lv, S.; Wang, Y.; Zhang, X.; Cai, Z.; Gu, W.; Li, Z.; Jiang, G.; Gao, F., Smart PdH@MnO(2) Yolk-Shell Nanostructures for Spatiotemporally Synchronous Targeted Hydrogen Delivery and Oxygen-Elevated Phototherapy of Melanoma. *ACS Nano* **2022,** *16* (4), 5597-5614.

9. Zhu, Y.; Wang, X.; Feng, L.; Zhao, R.; Yu, C.; Liu, Y.; Xie, Y.; Liu, B.; Zhou, Y.; Yang, P., Intermetallics triggering pyroptosis and disulfidptosis in cancer cells promote anti-tumor immunity. *Nat Commun* **2024,** *15* (1), 8696.
